# Safety and Immunogenicity of SARS-CoV-2 Spike Receptor-Binding Domain and N-Terminal Domain mRNA Vaccine

**DOI:** 10.1093/infdis/jiaf022

**Published:** 2025-01-10

**Authors:** Spyros Chalkias, Antionette Pragalos, Adebayo Akinsola, Gary Berman, Madhavi Ampajwala, Jay Meyer, Lorraine Schoch, Wen Zhou, Yamuna D Paila, Weiping Deng, Jing Feng, Elizabeth de Windt, Darin Edwards, Jacqueline Miller, Rituparna Das

**Affiliations:** Clinical Development, Infectious Diseases, Moderna, Inc, Cambridge, Massachusetts, USA; CTI Clinical Trial and Consulting Services, Covington, Kentucky, USA; Tekton Research, Atlanta, Georgia, USA; Clinical Research Institute, Inc, Minneapolis, Minnesota, USA; Advanced Care Research Centers (ACRC) Trials, Plano, Texas, USA; Velocity Clinical Research, Lincoln, Nebraska, USA; Clinical Operations, Infectious Disease, Moderna, Inc, Cambridge, Massachusetts, USA; Biostatistics, Moderna, Inc, Cambridge, Massachusetts, USA; Clinical Biomarkers, Infectious Disease, Moderna, Inc, Cambridge, Massachusetts, USA; Biostatistics, Moderna, Inc, Cambridge, Massachusetts, USA; Biostatistics, Moderna, Inc, Cambridge, Massachusetts, USA; Clinical Safety, Moderna, Inc, Cambridge, Massachusetts, USA; Infectious Disease, Moderna, Inc, Cambridge, Massachusetts, USA; Research and Development, Infectious Disease, Moderna, Inc, Cambridge, Massachusetts, USA; Research and Development, Infectious Disease, Moderna, Inc, Cambridge, Massachusetts, USA

**Keywords:** COVID-19, SARS-CoV-2, mRNA vaccines, mRNA-1283, mRNA-1273, booster vaccination, clinical study

## Abstract

**Background:**

mRNA-1283 is an investigational coronavirus disease 2019 (COVID-19) mRNA vaccine encoding the receptor-binding and N-terminal domains of the severe acute respiratory syndrome coronavirus 2 (SARS-CoV-2) spike protein in contrast to the original mRNA-1273 vaccine, which encodes the full-length spike protein.

**Methods:**

A phase 2a, dose-ranging, observer-blind, randomized study conducted in adults (aged ≥18 years) previously vaccinated with mRNA-1273 evaluated the safety and immunogenicity of a single dose of mRNA-1283 (2.5, 5, and 10 µg) and its bivalent formulation, mRNA-1283.211 (5 and 10 µg; encoding original SARS-CoV-2 and Beta) against the comparator mRNA-1273 vaccine, 50 µg (part A). A subsequent, open-label study (part B) evaluated the safety and immunogenicity of a monovalent Omicron BA.1 vaccine, mRNA-1283.529 (5 and 10 µg).

**Results:**

A total of 340 participants were randomized in part A, and 200 participants were enrolled in part B. All dose levels of mRNA-1283 vaccines were well tolerated and increased the neutralizing antibody (nAb) responses at day 29 compared to baseline against SARS-CoV-2 D614G and Beta. The nAb responses elicited by mRNA-1283 were higher than those elicited by mRNA-1273. mRNA-1283.529 (part B) increased nAb at day 29 against Omicron BA.1. Antibody responses remained detectable for a year postvaccination.

**Conclusions:**

mRNA-1283 was well tolerated and exhibited improved immunogenicity compared to mRNA-1273.

**Clinical Trials Registration:**

NCT05137236.

## Teaser

The mRNA-1283 vaccine encoding the RBD and NTD of the SARS-CoV-2 spike protein was well tolerated and had improved immunogenicity compared with mRNA-1273, regardless of the vaccine valency (monovalent, bivalent) or the variant sequence tested (SARS-CoV-2 D614G, Beta, Omicron BA.1).

Messenger RNA (mRNA)-based vaccines, including mRNA-1273 (Spikevax; Moderna, Inc), protect against coronavirus disease 2019 (COVID-19), including severe outcomes such as hospitalization and death [1–4]. While the adaptability of the mRNA platform enables rapid vaccine updates to address severe acute respiratory syndrome coronavirus 2 (SARS-CoV-2) variants [5, 6], the ultralow temperature storage conditions and limited refrigerated shelf life limit the flexibility of administration [7, 8].

The mRNA-1283 vaccine encodes 2 segments of the SARS-CoV-2 spike protein: the receptor-binding domain (RBD) and the N-terminal domain (NTD), thus incorporating a smaller mRNA molecule compared to vaccines that encode for the entire spike protein [6, 9]. The RBD and NTD are the immunodominant epitopes of the SARS-CoV-2 spike and are responsible for the majority of the anti-spike functional antibody responses that are considered protective [6, 9, 10]. Preclinical models indicate that mRNA-1283 induces increased antigen expression and similar or higher antibody responses compared with mRNA-1273 [6]. In a phase 1 study, a 2-dose primary series of mRNA-1283 (10 µg, 30 µg, and 100 µg) elicited functional antibody responses similar to a 2-dose regimen of mRNA-1273 (100 µg) [9]. In addition, a shorter length of mRNA is associated with increased mRNA stability [11], and the shorter sequence encoding the mRNA-1283 vaccine could have improved refrigerator stability compared with mRNA-1273 [6], which may facilitate the deployment of mRNA-1283 globally.

To further evaluate mRNA-1283, we conducted a phase 2a, randomized, observer-blind, dose-ranging study and used the original mRNA-1283 (encodes the original SARS-CoV-2 spike NTD and RBD) and a Beta bivalent formulation of mRNA-1283 (mRNA-1283.211) administered as a single dose. Subsequently, due to the emergence of Omicron variants in November 2021, we also included an open-label study part to evaluate a monovalent Omicron BA.1 mRNA-1283 vaccine candidate (mRNA-1283.529). Here, we present the safety and immunogenicity results of the study, which indicate that mRNA-1283 is well tolerated and has improved immunogenicity compared to mRNA-1273.

## METHODS

### Study Design and Participants

This was a phase 2a, two-part, dose-ranging study conducted at 16 sites in the United States to evaluate the safety, reactogenicity, and immunogenicity of the mRNA-1283 vaccine and its variant-containing formulations, mRNA-1283.211 and mRNA-1283.529, in healthy adults previously vaccinated with mRNA-1273 (ClinicalTrials.gov identifier, NCT05137236).

Part A was an observer-blind, stratified, randomized study evaluating healthy adults (aged ≥18 years) who received a 2-dose mRNA-1273 primary series (100 µg) ≥ 6 months before screening and enrollment. Participants were randomly assigned (1:1:1:1:1:1) using an interactive response technology to receive a single dose of mRNA-1283 (2.5 µg, 5 µg, or 10 µg), mRNA-1283.211 (5 µg or 10 µg), or mRNA-1273 (50 µg) (Supplementary Figure 1).

Part B was an open-label study that enrolled adults (aged ≥18 years) who had received a 2-dose mRNA-1273 primary series (100 µg) followed by an mRNA-1273 booster (50 µg) ≥ 3 months before screening and enrollment. Participants were enrolled (1:1) and received a single dose of mRNA-1283.529 (5 µg or 10 µg) (Supplementary Figure 1). Full eligibility criteria are provided in the Supplementary Material.

Enrollment in both study parts was stratified by age (18–55 and ≥56 years), with ≥20% but ≤50% of participants ≥56 years of age. Participants received study vaccine on day 1 and were followed for 12 months. All participants were tested for SARS-CoV-2 infection on days 1, 29, 181, and 366, and any symptoms of COVID-19 triggered additional testing visits. Breakthrough COVID-19 cases were monitored by active surveillance throughout the study and were reported as clinical events.

The study was conducted in accordance with the consensus ethical principles derived from international guidelines, including the Declaration of Helsinki. The protocol and study documents were approved by the institutional review board before the start of the study. Written informed consent was obtained by all participants before performing study procedures.

### Vaccines

The mRNA-1283 vaccine contains a single mRNA sequence encoding the RBD and NTD segments of the original SARS-CoV-2 spike protein linked together with a flexible linker and anchored to a transmembrane domain from influenza hemagglutinin. Bivalent mRNA-1283.211 encodes the RBD and NTD of the original SARS-CoV-2 and Beta spike proteins in a 1:1 coformulation. The mRNA-1273 active comparator (used in part A only) contains a single mRNA sequence encoding the full-length prefusion-stabilized spike glycoprotein of the original SARS-CoV-2. Monovalent mRNA-1283.529 encodes the RBD and NTD of Omicron BA.1 spike protein. Vaccines were stored at −90°C to −60°C (mRNA-1283, mRNA-1283.211, and mRNA-1283.529) and −25°C to −15°C (mRNA-1273). mRNA-1273 was provided as a sterile solution for injection at a concentration of 0.2 mg/mL. mRNA-1283 or mRNA-1283.211 injection contained 0.4 mg/mL of total RNA in an RNA-lipid nanoparticle (LNP) dispersion, while mRNA-1283.529 injection contained 0.1 mg/mL of total RNA in an RNA-LNP dispersion. All vaccines were administered at a volume of 0.25 mL into the deltoid muscle.

### Objectives

The primary objectives in part A were to assess the reactogenicity, safety, and immunogenicity of a single booster dose of an mRNA-1283 (2.5 µg, 5 µg, and 10 µg) and a Beta bivalent vaccine mRNA-1283.211 (5 µg and 10 µg). Immune responses against SARS-CoV-2 D614G, Beta, as well as Omicron BA.1 were assessed through day 29 as the primary objective and at all time points as the secondary objective.

The primary objectives in part B were to assess the safety, reactogenicity, and immunogenicity of a single booster dose of a monovalent Omicron BA.1 vaccine mRNA-1283.529 (5 µg and 10 µg). Immune responses against SARS-CoV-2 D614G and Omicron BA.1 were assessed through day 29 as the primary objective, and at all time points as the secondary objective.

Active surveillance for breakthrough SARS-CoV-2 infections and COVID-19 cases occurred throughout the study in both parts A and B, and was evaluated as an exploratory objective (Supplementary Material).

### Safety Assessments

Reactogenicity assessments in parts A and B included the frequency and grade of solicited local and systemic adverse reactions (ARs; recorded daily in electronic diaries using a structured checklist) through 7 days after vaccination (Supplementary Table 1). Safety assessments (parts A and B) included unsolicited treatment-emergent AEs (TEAEs) through 28 days after vaccination; and any serious AEs (SAEs), medically attended AEs (MAAEs; AEs leading to an unscheduled visit to a health care professional), AEs leading to study participation withdrawal, and AEs of special interest (AESIs; serious or nonserious AEs of concern for which ongoing monitoring and immediate notification by the investigator to the trial sponsor is required) from day 1 to end of study.

### Immunogenicity Assessments

Blood samples for immunogenicity assessments in parts A and B were collected at scheduled visits on days 1, 29, 91, 181, and 366. Serum neutralizing antibody (nAb) titers were assessed using a validated SARS-CoV-2 spike (S)-pseudotyped virus neutralization assay in 293/ACE2 cells against SARS-CoV-2 D614G, Beta, and Omicron BA.1 (Duke Laboratories) [12–14] (Supplementary Material). Active surveillance for intercurrent or breakthrough SARS-CoV-2 infections was conducted throughout the study. All participants were tested for the presence of SARS-CoV-2 anti-nucleocapsid antibodies at days 1, 29, 181, and 366, as well as by nasopharyngeal swab reverse transcriptase polymerase chain reaction (RT-PCR) on days 1, 29, 181, and 366 (Supplementary Material).

### Statistical Analyses

The sample size in this dose-ranging study was not driven by statistical assumptions for formal statistical testing, and the number of randomized participants was considered sufficient to provide a descriptive summary of the safety and immunogenicity of each vaccine group.

Safety analyses were based on the safety set (all participants who received a study vaccine), with the exception of the analysis of solicited ARs that was based on the solicited safety set (all participants in the safety set who contributed any solicited AR data). Safety and reactogenicity were descriptively analyzed.

The per-protocol immunogenicity set (PPIS) included all participants who received the planned study vaccine, including participants with and without prior SARS-CoV-2 infection, with prebooster and day 29 nAb data, no previous HIV infection, and no major protocol deviations that impacted immunogenicity data. The primary analysis of immunogenicity was conducted using the PPIS-negative (participants in the PPIS who were negative for SARS-CoV-2 before the booster, as defined in the Supplementary Material). Prespecified analyses of immunogenicity were also performed using the PPIS.

In both parts A and B, immunogenicity against SARS-CoV-2 D614G, Beta, and Omicron BA.1 at each time point was assessed using geometric mean titer (GMT), geometric mean fold rise (GMFR), and seroresponse rate. GMT and GMFR were determined alongside corresponding 95% confidence intervals (CIs), which were calculated based on the *t* distribution of the log-transformed values, then back-transformed to the original scale. Seroresponse rates (defined in the Supplementary Material) were provided with 95% CIs calculated using the Clopper-Pearson method.

In part A, the primary immunogenicity endpoints of SARS-CoV-2–specific nAb levels against SARS-CoV-2 D614G, Beta, and Omicron BA.1 were assessed for mRNA-1283 and mRNA-1283.211 relative to mRNA-1273 using an analysis of covariance (ANCOVA) model. Day 29 serum nAb titers against SARS-CoV-2 D614G, Beta, or Omicron BA.1 were included as a dependent variable, and the vaccine group was included as a fixed effect, adjusting the model for age group (18–55 years, ≥ 56 years) and prior SARS-CoV-2 infection as appropriate. GMTs and GMFRs were estimated using geometric least square means from the model and were summarized for each dose of mRNA-1283 and mRNA-1283.211 with 95% CIs. The ratios of GMTs (mRNA-1283 and mRNA-1283.211 [each dose] relative to mRNA-1273) were estimated using the ratios of geometric least square means and summarized with 95% CIs. In part B, GMTs and GMFRs for nAbs against SARS-CoV-2 D614G and Omicron BA.1 were summarized with 95% CIs using the same methods as described for part A. For parts A and B, the numbers and percentages of asymptomatic and symptomatic SARS-CoV-2 infections and COVID-19 cases were summarized using the full analysis set.

## RESULTS

### Study Population and Baseline Characteristics

In part A, between 6 December 2021 and 17 February 2022, a total of 340 participants who had previously completed a 2-dose primary series of mRNA-1273 (100 µg) were randomly assigned to receive 1 dose of mRNA-1283 (2.5 µg, n = 57; 5 µg, n = 63; or 10 µg, n = 56), mRNA-1283.211 (5 µg, n = 53 or 10 µg, n = 54), or mRNA-1273 (50 µg, n = 57) (Supplementary Figure 2*A*). The age across groups (randomized set) ranged from 19 to 87 years; 54.4% to 72.2% of participants were female, 68.5% to 82.5% were White, and 46.3% to 65.1% had no evidence of SARS-CoV-2 infection at baseline (Table 1). The median interval from the last dose (second dose of primary series) ranged from 8.4 to 9.0 months (Table 1).

**Table 1.** Baseline Characteristics of Participants in Parts A (Randomized Set) and B (Enrolled Set)

Characteristic	Part A						Part B	
	mRNA-1283			mRNA-1283.211		mRNA-1273	mRNA-1283.529	
	2.5 µg (n = 57)	5 µg (n = 63)	10 µg (n = 56)	5 µg (n = 53)	10 µg (n = 54)	50 µg (n = 57)	5 µg (n = 103)	10 µg (n = 97)
Age, y								
n	57	63	56	53	54	57	103	97
Mean ± SD	44.6 ± 15.1	45.7 ± 14.8	44.5 ± 14.2	45.8 ± 15.8	47.1 ± 12.6	44.2 ± 13.8	57.3 ± 14.3	52.1 ± 14.2
Median (range)	42 (19–79)	45 (19–74)	44 (21–79)	47 (19–87)	46 (19–79)	41 (23–75)	59 (21–93)	50 (19–85)
18–55 y, n (%)	40 (70.2)	45 (71.4)	42 (75.0)	38 (71.7)	40 (74.1)	42 (73.7)	44 (42.7)	59 (60.8)
≥56 y, n (%)	17 (29.8)	18 (28.6)	14 (25.0)	15 (28.3)	14 (25.9)	15 (26.3)	59 (57.3)	38 (39.2)
Sex, n (%)								
Male	26 (45.6)	20 (31.7)	24 (42.9)	19 (35.8)	15 (27.8)	25 (43.9)	33 (32.0)	35 (36.1)
Female	31 (54.4)	43 (68.3)	32 (57.1)	34 (64.2)	39 (72.2)	32 (56.1)	70 (68.0)	62 (63.9)
Race, n (%)								
White	41 (71.9)	47 (74.6)	41 (73.2)	39 (73.6)	37 (68.5)	47 (82.5)	94 (91.3)	88 (90.7)
Black or African American	11 (19.3)	11 (17.5)	9 (16.1)	9 (17.0)	10 (18.5)	6 (10.5)	7 (6.8)	7 (7.2)
Asian	5 (8.8)	1 (1.6)	3 (5.4)	3 (5.7)	3 (5.6)	3 (5.3)	0	2 (2.1)
Native Hawaiian or Other Pacific Islander	0	0	0	0	0	0	1 (1.0)	0
Ethnicity, n (%)								
Hispanic or Latino	10 (17.5)	13 (20.6)	8 (14.3)	10 (18.9)	8 (14.8)	11 (19.3)	2 (1.9)	4 (4.1)
Not Hispanic or Latino	47 (82.5)	50 (79.4)	47 (83.9)	43 (81.1)	43 (79.6)	45 (78.9)	100 (97.1)	93 (95.9)
SARS-CoV-2 infection status at baseline,^a^ n (%)								
Negative	31 (54.4)	41 (65.1)	34 (60.7)	30 (56.6)	25 (46.3)	29 (50.9)	77 (74.8)	71 (73.2)
Positive	26 (45.6)	21 (33.3)	22 (39.3)	23 (43.4)	29 (53.7)	26 (45.6)	24 (23.3)	26 (26.8)
Missing	0	1 (1.6)	0	0	0	2 (3.5)	2 (1.9)	0
Interval from prior dose,^b,c^ mo								
n	50	52	49	45	46	53	103	92
Mean ± SD	8.8 ± 1.5	8.9 ± 1.4	9.0 ± 1.4	8.5 ± 2.0	8.7 ± 1.3	9.1 ± 1.6	4.7 ± 1.9	4.7 ± 2.4
Median (range)	8.8 (3.1–11.1)	9.0 (6.2–12.1)	8.8 (6.2–11.8)	8.4 (2.1–12.2)	8.5 (6.2–11.5)	9.0 (6.1–12.7)	4.1 (2.4–13.1)	4.0 (3.0–17.4)

Numbers are based on planned vaccine group and percentages are based on the number of randomized participants.

Abbreviations: COVID-19, coronavirus disease 2019; mo, month; mRNA, messenger RNA; RT-PCR, reverse transcription polymerase chain reaction; SARS-CoV-2, severe acute respiratory syndrome coronavirus 2.

^a^Prebooster/baseline SARS-CoV-2 status: positive was defined by immunologic or virologic evidence of prior COVID-19 (positive RT-PCR test or positive Elecsys result at day 1); negative status was defined as negative RT-PCR test and negative Elecsys result at day 1.

^b^Boosting interval for part A was defined as time duration from second dose of primary series to booster dose (months) = (booster dose day− second dose day of primary series + 1)/30.4375. The last dose date reported by each participant prior to the study was used for this calculation.

^c^Boosting interval for part B was defined as time duration from first booster dose to second booster dose (months) = (second booster dose day − first booster dose day + 1)/30.4375. The last dose date reported by each participant prior to the study was used for this calculation.

In part B, between 23 February 2022 and 15 March 2022, a total of 200 participants who had received a primary series and a booster dose of mRNA-1273 were enrolled to receive a second booster dose of mRNA-1283.529 (5 µg, n = 103; 10 µg, n = 97) (Supplementary Figure 2*B*). Across the mRNA-1283.529 groups (5 µg, 10 µg), the age range was similar (21–93 and 19–85 years, respectively), as was the percentage of women (68.0% and 63.9%), White participants (91.3% and 90.7%), and participants with no evidence of SARS-CoV-2 infection at baseline (prebooster) (74.8% and 73.2%) (Table 1). The median interval from the last dose (first mRNA-1273 booster dose, 50 µg) was 4 months for both groups (Table 1).

### Safety and Reactogenicity

**Figure 1.**
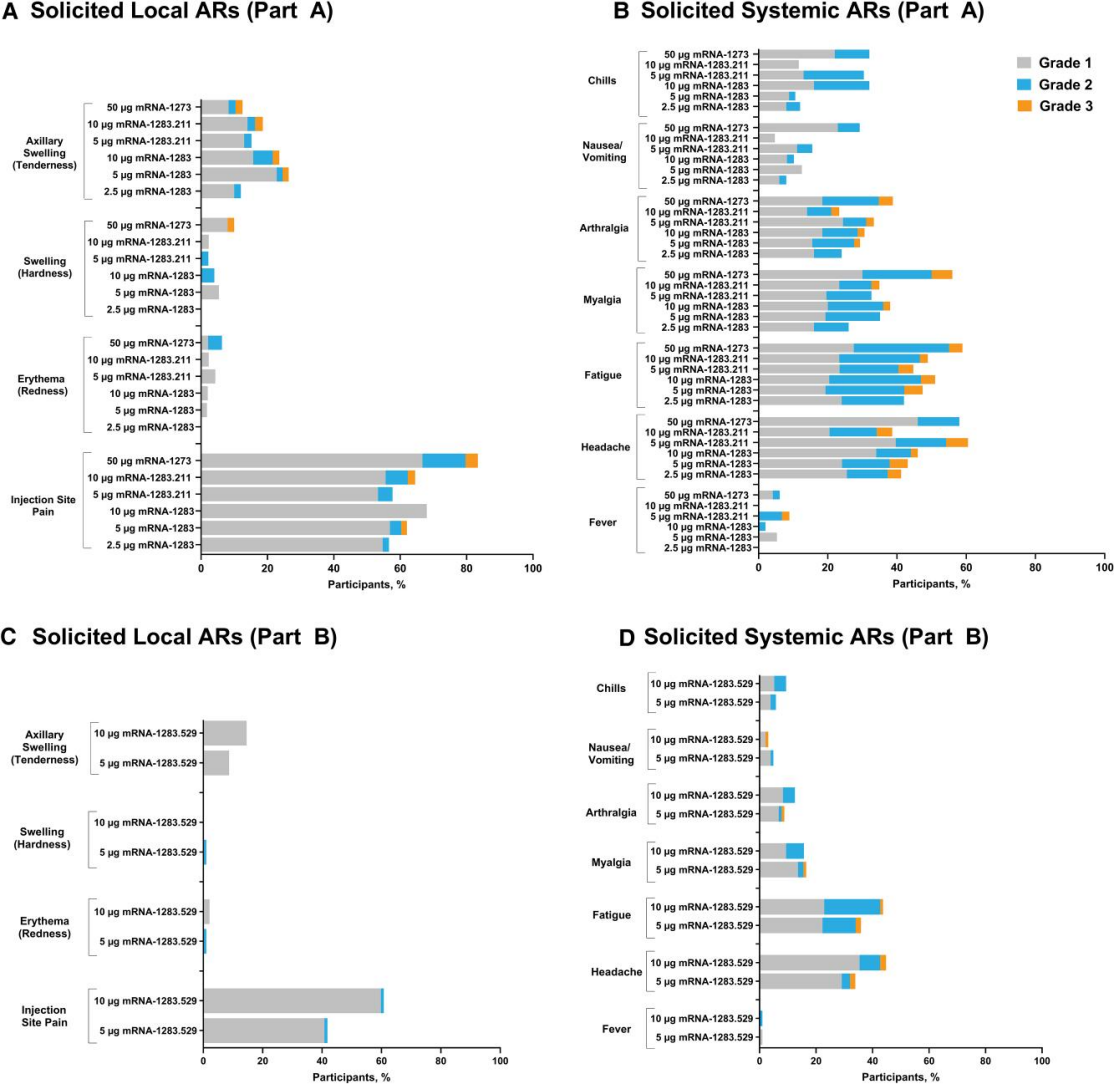
Participant incidence of solicited local and systemic ARs in parts A and B (solicited safety set). *A*, Solicited local adverse reactions (part A) after each booster dose of mRNA-1283 (2.5 µg, 5 µg, and 10 µg), Beta bivalent mRNA-1283.211 (5 µg and 10 µg), and mRNA-1273 (50 µg). *B*, Solicited systemic adverse reactions (part A) after each booster dose of mRNA-1283 (2.5 µg, 5 µg, and 10 µg), Beta bivalent mRNA-1283.211 (5 µg and 10 µg), and mRNA-1273 (50 µg). *C*, Solicited local adverse reactions (part B) after a single booster dose of monovalent Omicron BA.1 mRNA-1283.529 (5 µg and 10 µg). *D*, Solicited systemic adverse reactions (part B) after a single booster dose of monovalent Omicron BA.1 mRNA-1283.529 (5 µg and 10 µg). The local ARs that were solicited by the electronic diary in parts A and B included pain at injection site, erythema (redness) at injection site, swelling (hardness) at injection site, localized axillary swelling or tenderness ipsilateral to the injection arm, and swelling or tenderness (groin or underarm) ipsilateral to the side of injection. The systemic ARs that were solicited by the electronic diary in parts A and B included headache, fatigue, myalgia (muscle aches all over the body), arthralgia (aching in several joints), nausea/vomiting, fever, and chills. The solicited safety set included all participants in the safety set (all randomly assigned [part A]/enrolled [part B] participants who received study vaccine, analyzed based on the study vaccine received) who contributed any solicited AR data; the solicited safety set comprised 314 (92.4%) participants in part A and 199 (99.5%) participants in part B. Abbreviations: AR, adverse reaction; mRNA, messenger RNA.

In part A, the incidence of participants with any solicited local ARs was numerically lower in the mRNA-1283 (range, 56.6% [30/53] to 72.5% [37/51]) and mRNA-1283.211 groups (5 µg, 58.3% [28/48]; 10 µg, 63.0% [29/46]) compared to the mRNA-1273 group (85.2% [46/54]). The majority of solicited local ARs were grade 1 followed by grade 2, with no grade 4 events reported (Figure 1). The incidence of grade 3 solicited local ARs was 0 in the 2.5-µg mRNA-1283 and 5-µg mRNA-1283.211 groups and ranged between 1.7% and 2.2% (1 participant in each group) across the remaining mRNA-1283 and mRNA-1283.211 groups; in the mRNA-1273 group, the incidence of grade 3 solicited local ARs was 5.6%. The most common solicited local AR was injection site pain. The incidence of participants with any solicited systemic ARs was similar or lower in the mRNA-1283 dose groups (range, 62.7% [32/51] to 68.6% [35/51]) compared to the mRNA-1273 group (77.4% [41/53]) (Figure 1). For the mRNA-1283.211 dose groups, incidence rates were overall similar to that of the mRNA-1273 group. The majority of solicited systemic ARs were grades 1 or 2. The incidence of grade 3 solicited systemic ARs ranged from 3.9% (2/51) to 8.5% (5/59) in the mRNA-1283 and mRNA-1283.211 groups, respectively, and was 9.4% (5/53) in the mRNA-1273 group. No grade 4 solicited systemic ARs were reported. Across vaccine groups, headache and fatigue were the most commonly reported solicited systemic ARs.

The incidence of participants reporting unsolicited TEAEs through 28 days after vaccination in part A was similar across the vaccine groups; no deaths, AESIs, or AEs leading to discontinuation from the study were reported (Supplementary Table 2). The participant incidence of unsolicited TEAEs, severe TEAEs, SAEs, MAAEs, and AESIs assessed throughout the study was similar among mRNA-1283, mRNA-1283.211, and mRNA-1273 groups. There were no fatal AEs, and no AEs led to study discontinuation. Per investigator assessment, none of the SAEs or AESIs were considered vaccine-related; 1 MAAE in the mRNA-1273 group was considered vaccine-related (nonserious joint swelling). No participants experienced anaphylaxis, and no events of myocarditis or pericarditis were reported through the end of the study.

In part B, the incidence of participants with any solicited local ARs was 71.9% (69/96) in the 10-µg mRNA-1283.529 group and 44.7% (46/103) in the 5-µg mRNA-1283.529 group. The majority of solicited local ARs were grade 1 (mRNA-1283.529, 5 µg, 41.7%; 10 µg, 70.8%) followed by grade 2 (mRNA-1283.529, 5 µg, 2.9%; 10 µg, 1.0%), with no grade 3 or 4 events reported (Figure 1). The most commonly reported solicited local AR was injection site pain. The incidence of participants reporting any systemic AR was 62.5% (60/96) in the 10-µg mRNA-1283.529 group and 49.5% (51/103) in the 5-µg mRNA-1283.529 group. The majority of solicited systemic ARs were grade 1 (mRNA-1283.529, 5 µg, 33.0%; 10 µg, 36.5%) followed by grade 2 (mRNA-1283.529, 5 µg, 13.6%; 10 µg, 21.9%), with no grade 4 events reported (Figure 1). The participant incidence of grade 3 solicited systemic ARs was 2.9% (3/103) and 4.2% (4/96) in the 5-µg and 10-µg mRNA-1283.529 groups, respectively. The most commonly reported solicited systemic ARs were headache and fatigue.

The incidence of participants reporting unsolicited TEAEs through 28 days in part B was similar between the mRNA-1283.529 groups; no deaths, SAEs, AESIs, or AEs leading to study discontinuation were reported (Supplementary Table 3). The participant incidence of unsolicited TEAEs, severe TEAEs, and MAAEs through the end of study was similar between vaccine groups. SAEs were reported in 1.9% (2/103) and 6.2% (6/97) of participants in the 5-µg and 10-µg mRNA-1283.529 groups, respectively. Of participants reporting SAEs, 2 (2.1%) had fatal outcomes (both in the 10-µg mRNA-1283.529 group); none were considered vaccine-related by the investigator. One participant with a fatal outcome was a 40-year-old man with a history of hypertension and increased blood cholesterol, who died of a myocardial infarction due to hypertensive heart disease and atherosclerosis 3 months after vaccine administration. The other participant with a fatal outcome was a 50-year-old woman with a history of hypercholesterolemia, depression, anxiety, insomnia, Sjogren syndrome, and Ehlers-Danlos syndrome, who died at home due to an unknown cause approximately 3 months after vaccine administration. AESIs were reported only in the 10-µg mRNA-1283.529 group (3.1% [3/97]); no events of myocarditis, pericarditis, or anaphylaxis were reported. Throughout the study, none of the SAEs, AEs leading to study discontinuation, or AESIs were considered vaccine-related by the investigator.

### Immunogenicity

**Figure 2.**
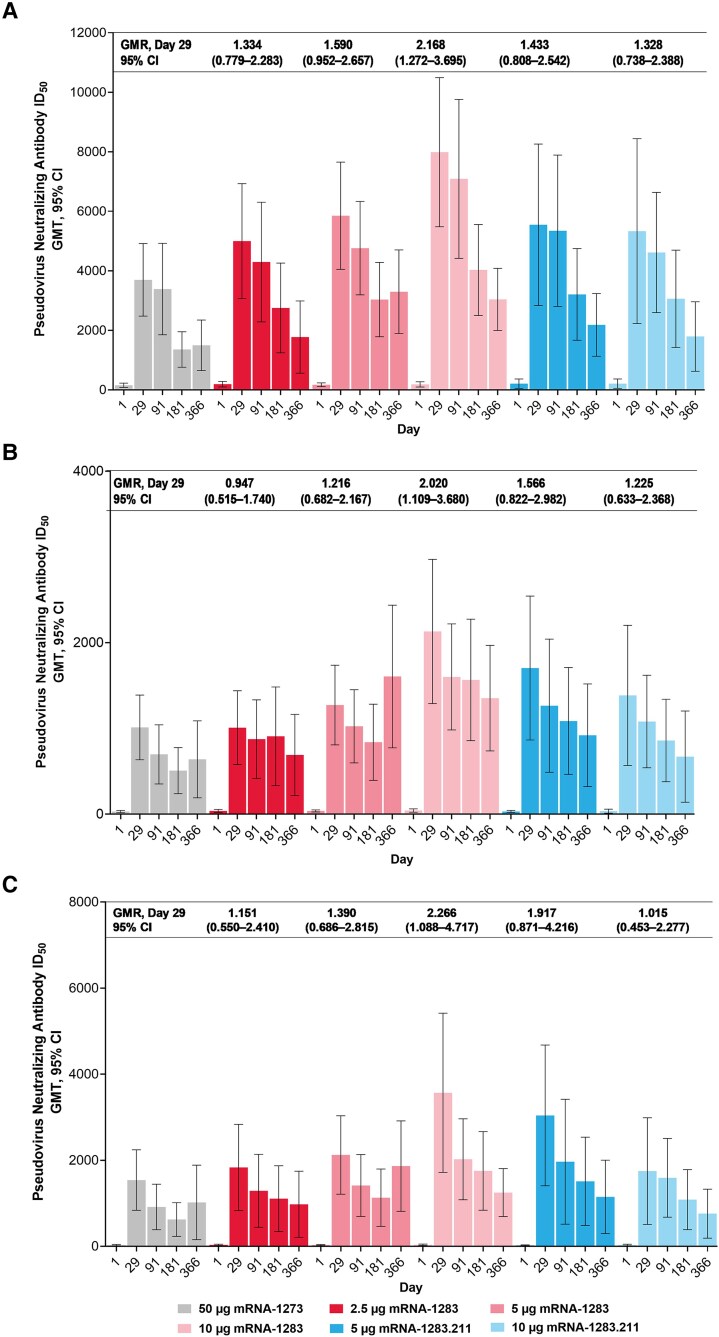
Serum nAb responses to SARS-CoV-2 D614G, Beta, and Omicron BA.1 in study part A. Serum nAb titers (part A; PPIS-negative) on days 1 (baseline), 29, 91, 181, and 366 after single booster doses of mRNA-1283 (2.5 µg, 5 µg, and 10 µg), Beta bivalent mRNA-1283.211 (5 µg and 10 µg), or mRNA-1273 (50 µg) against (*A*) SARS-CoV-2 D614G, (*B*) Beta, and (*C*) Omicron BA.1. Antibody values reported as below the LLOQ were replaced by 0.5 × LLOQ. Values greater than the ULOQ were converted to the ULOQ if actual values were not available. The 95% CIs were calculated based on the *t* distribution of the log-transformed values or the difference in the log-transformed values for GMT, geometric mean fold rise, respectively, then back-transformed to the original scale for presentation. The PPIS-negative population consisted of all randomly assigned participants who received the planned study vaccine, were negative for SARS-CoV-2 before the booster dose, had prebooster and day 29 nAb data, no previous HIV infection, and no major protocol deviations that impacted key/critical data; the PPIS-negative population consisted of 175 (51.5%) participants in part A. Abbreviations: CI, confidence interval; GMT, geometric mean titer; ID_50_, 50% inhibitory dilution; LLOQ, lower limit of quantification; mRNA, messenger RNA; nAb, neutralizing antibody; PPIS, per-protocol immunogenicity set; SARS-CoV-2, severe acute respiratory syndrome coronavirus 2; ULOQ, upper limit of quantification.

In participants without a prior infection (primary analysis set; PPIS-negative) in part A, all dose levels of mRNA-1283, mRNA-1283.211, and mRNA-1273 increased nAb responses at day 29 relative to baseline against SARS-CoV-2 D614G, Beta, and Omicron BA.1; the nAb responses were generally higher in the mRNA-1283 groups compared to the mRNA-1273 group (Figure 2 and Supplementary Table 4). In the ANCOVA model of nAb titers (PPIS-negative) adjusting for age groups, the GMR of mRNA-1283 groups versus mRNA-1273 at day 29 favored mRNA-1283 for each variant tested, except for the GMR of mRNA-1283 2.5 µg versus mRNA-1273 against Beta (Figure 2 and Supplementary Table 5). nAb titers remained detectable on days 91, 181, and 366 in all vaccine groups (Figure 2 and Supplementary Tables 4 and 5). The nAb responses were consistent in the analysis of the overall immunogenicity population (all participants in the immunogenicity set regardless of prior infection; PPIS) (Supplementary Tables 6 and 7).

**Figure 3.**
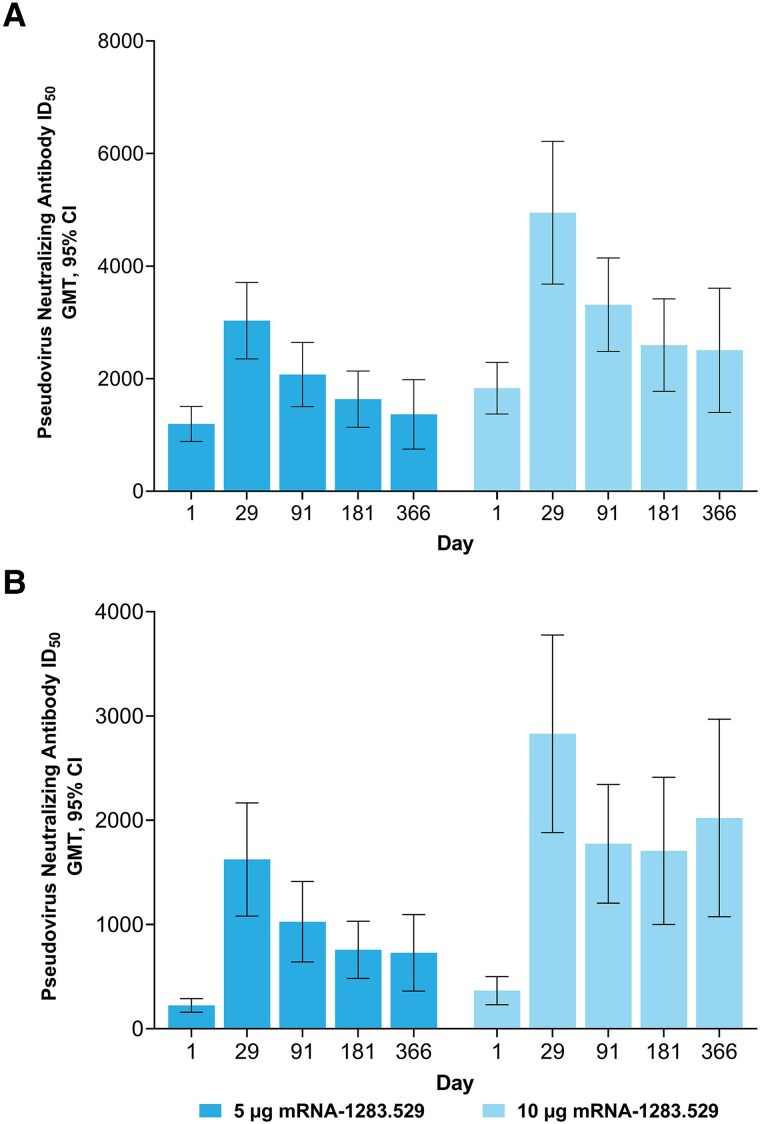
Serum nAb responses to SARS-CoV-2 D614G and Omicron BA.1 in study part B. Serum nAb titers (part B, PPIS-negative) on days 1 (baseline), 29, 91, 181, and 366 after single booster doses of mRNA-1283.529 (5 µg and 10 µg) against (*A*) SARS-CoV-2 D614G and (*B*) Omicron BA.1. Antibody values reported as below the LLOQ were replaced by 0.5 × LLOQ. Values greater than the ULOQ were converted to the ULOQ if actual values were not available. The 95% CIs were calculated based on the *t* distribution of the log-transformed values or the difference in the log-transformed values for GMT, geometric mean fold rise, respectively, then back-transformed to the original scale for presentation. The PPIS-negative population consisted of 144 (72.0%) participants in part B. Abbreviations: CI, confidence interval; GMT, geometric mean titer; ID_50_, 50% inhibitory dilution; LLOQ, lower limit of quantification; mRNA, messenger RNA; nAb, neutralizing antibody; PPIS, per-protocol immunogenicity set; SARS-CoV-2, severe acute respiratory syndrome coronavirus 2; ULOQ, upper limit of quantification.

In part B, nAb increases relative to baseline at day 29 (PPIS-negative) were observed in the 5-µg and 10-µg mRNA-1283.529 groups against SARS-CoV-2 D614G and Omicron BA.1 (Figure 3 and Supplementary Table 8). The nAb responses remained detectable through days 91, 181, and 366 (Figure 3 and Supplementary Table 8). A similar trend was observed in the PPIS population (Supplementary Table 9).

Lastly, although this study was not designed to evaluate vaccine efficacy, the frequencies of SARS-CoV-2 infections and COVID-19 events were balanced across vaccine groups in part A (Supplementary Table 10) and part B (Supplementary Table 11).

## DISCUSSION

Authorized mRNA COVID-19 vaccines encode the entire spike protein of SARS-CoV-2 [15–17] and are highly efficacious in protecting against COVID-19 [18]. The major antigenic domains of the spike protein, the RBD and NTD, are known to be immunodominant sites responsible for the nAb responses that are considered protective against COVID-19 [10, 19, 20]. By contrast, the spike regions outside these domains are associated with nonneutralizing responses that are broadly cross-reactive to endemic coronaviruses [21]. mRNA-1283 is designed to target only the RBD and NTD, and animal studies indicate that it enhances protein expression for these 2 domains and provides protection against respiratory disease [6]. In addition, mRNA-1283 administered on a 2-dose schedule at a dose level of 10 µg elicited immune responses similar to the 2-dose primary series of mRNA-1273 (100 µg) in a phase 1 clinical study [9]. Therefore, in the present phase 2a study, we evaluated mRNA-1283 administered as a single dose of 10 µg and included even lower dose levels of 5 µg and 2.5 µg.

The mRNA-1283 (2.5 µg, 5 µg, and 10 µg), the Beta bivalent mRNA-1283.211 (5 µg and 10 µg), and the Omicron BA.1 monovalent mRNA-1283.529 (5 µg and 10 µg) vaccines increased nAb responses against SARS-CoV-2 D614G, Beta, and Omicron BA.1. Specifically, in the randomized part of the study (part A, mRNA-1283 and mRNA-1283.211), nAb responses elicited by mRNA-1283 were generally higher compared to mRNA-1273 (50 µg) at day 29 against all variants tested, consistent with the potential of mRNA-1283 to enhance immune responses [6]. In part B, the nAb responses against Omicron BA.1 generated by a single dose of mRNA-1283.529 were consistent with those elicited by the Omicron BA.1-encoding mRNA-1273 vaccine in separate clinical studies [12, 22]. The nAb responses elicited by mRNA-1283 persisted through 1 year, although antibody titers gradually decreased. The safety profile of mRNA-1283 (all dose levels) was similar to the mRNA-1273 50-µg comparator, and no new safety concerns were identified.

This study was not designed or powered to evaluate vaccine efficacy, and there was no randomization in the open-label study part (part B). Although surveillance for COVID-19 was conducted, the vaccine groups were small and the study coincided with the first Omicron variant wave; infections in study participants likely reflect the epidemiological environment at the time. It is possible that breakthrough SARS-CoV-2 infections influenced the nAb responses, especially in the latter part of the study (6–12 months of follow-up).

mRNA-1283 was well tolerated and demonstrated improved immunogenicity compared to mRNA-1273, regardless of the vaccine valency (monovalent, bivalent) or the variant sequence tested (SARS-CoV-2 D614G, Beta, Omicron BA.1). The 10-µg dose level of mRNA-1283 achieved a balance between safety and immunogenicity and is expected to broaden vaccine supply through efficient production scaling. mRNA-1283 (10 µg) is being evaluated in a phase 3 pivotal study (NCT05815498), and, if approved, is expected to be updated for SARS-CoV-2 variants similarly to mRNA-1273. In addition, given that lower dose levels of mRNA-1283 elicit higher immune responses compared with mRNA-1273, mRNA-1283 provides a solid basis for the development of both stand-alone COVID-19 vaccines and combination vaccines targeting multiple respiratory pathogens.

## Supplementary Material

jiaf022_Supplementary_Data

## Supplementary Data

Supplementary materials are available at *The Journal of Infectious Diseases* online (http://jid.oxfordjournals.org/). Supplementary materials consist of data provided by the author that are published to benefit the reader. The posted materials are not copyedited. The contents of all supplementary data are the sole responsibility of the authors. Questions or messages regarding errors should be addressed to the author.

## Notes

***Author contributions.*** S. C., L. S., W. D., J. F., D. E., J. M., and R. D. conceived and planned the study. S. C., A. P., A. A., G. B., M. A., J. M., L. S., W. D., J. F., and R. D. collected the data. S. C., G. B., L. S., W. Z., W. D., J. F., E. d. W., D. E., Y. D. P., J. M., and R. D. contributed to data analysis and interpretation. S. C., A. P., A. A., G. B., M. A., J. M., L. S., W. Z., W. D., J. F., E. d. W., D. E., Y. D. P., J. M., and R. D. drafted the manuscript and contributed to the critical revision of the manuscript for important intellectual content. All the authors met the authorship criteria and approved the publication.

***Acknowledgments.*** Medical writing and editorial assistance were provided by Anja Varjačić, PhD, and Renee Gordon, PhD, of MEDiSTRAVA in accordance with Good Publication Practice (GPP 2022) guidelines and under the direction of the authors.

***Financial support*.** This work was supported by Moderna, Inc.

***Data availability.*** Access to patient-level data presented in this article and supporting clinical documents with external researchers who provide methodologically sound scientific proposals will be available upon reasonable request for products or indications that have been approved by regulators in the relevant markets and subject to review from 24 months after study completion. Such requests can be made to Moderna Inc, 325 Binney Street, Cambridge, MA 02142; data_sharing@modernatx.com. A materials transfer and/or data access agreement with the sponsor will be required for accessing shared data. All other relevant data are presented in the paper.
