# Supplementary material for: Safety and Immunogenicity of SARS-CoV-2 Spike Receptor-Binding Domain and N-Terminal Domain mRNA Vaccine
Source: J Infect Dis. 2025 Jan 10;231(4):e754–63. doi: 10.1093/infdis/jiaf022 (PMC11998576; doi:10.1093/infdis/jiaf022)
Supplement: jiaf022_Supplementary_Data [file jiaf022_supplementary_data.docx]

# Supplementary Information

## Inclusion/Exclusion Criteria

### Inclusion Criteria (Parts A and B)

Each participant had to fulfill all of the following criteria to be enrolled in the study:

1. At least 18 years of age at the time of consent (Screening Visit).
2. Investigator assessment that participant understands and is willing and physically able to comply with protocol-mandated follow-up, including all procedures.
3. Participant has provided written informed consent for participation in this study, including all evaluations and procedures as specified in this protocol.
4. Female participants of nonchildbearing potential may be enrolled in the study. Nonchildbearing potential is defined as bilateral tubal ligation >1 year prior to screening, bilateral oophorectomy, hysterectomy, or menopause. Follicle-stimulating hormone level may be measured at the discretion of the investigator to confirm postmenopausal status.
5. Female participants of childbearing potential may be enrolled in the study if the participant fulfills all the following criteria:
   1. Has a negative pregnancy test on the day of vaccination (Day 1).
   2. Has practiced adequate contraception or has abstained from all activities that could result in pregnancy for at least 28 days prior to Day 1.
   3. Has agreed to continue adequate contraception through 3 months following the last vaccine administration.
   4. Is not currently breastfeeding.
   5. Adequate female contraception is defined as consistent and correct use of a US Food and Drug Administration (FDA)–approved contraceptive method in accordance with the product label.
6. Participant must have received their second dose of the mRNA-1273 primary series ≥6 months prior to screening and enrollment (Part A) or have received the mRNA-1273 series and an mRNA-1273 booster dose (50 ug) ≥3 months prior to screening and enrollment (Part B).

### Exclusion Criteria (Parts A and B)

Participants meeting any of the following criteria were excluded from the study:

1. Had significant exposure to someone with SARS-CoV-2 infection or COVID-19 in the past 14 days, defined as a close contact of someone who had COVID-19.
2. Is acutely ill or febrile (temperature ≥ 38.0°C/100.4°F) less than 72 hours prior to or at the Screening Visit or Day 1. Participants meeting this criterion may be rescheduled and will retain their initially assigned participant number.
3. Currently has symptomatic acute or unstable chronic disease requiring medical or surgical care, to include significant change in therapy or hospitalization for worsening disease, at the discretion of the investigator.
4. Has a medical, psychiatric, or occupational condition that may pose additional risk as a result of participation, or that could interfere with safety assessments or interpretation of results according to the investigator’s judgment.
5. History of myocarditis, pericarditis, or myopericarditis within 2 months prior to Screening. Participants who have not returned to baseline after their convalescent period will also be excluded.
6. Has a current or previous diagnosis of immunocompromising condition to include human immunodeficiency virus, immune-mediated disease requiring immunosuppressive treatment, or other immunosuppressive condition.
7. Has received systemic immunosuppressants or immune-modifying drugs for >14 days in total within 6 months prior to screening (for corticosteroids ≥ 10 mg/day of prednisone equivalent) or is anticipating the need for immunosuppressive treatment at any time during participation in the study.
8. Has received or plans to receive any licensed vaccine ≤ 28 days prior to the injection (Day 1) or plans to receive a licensed vaccine within 28 days before or after the study injection, with the exception of influenza vaccines, which may be given 14 days before or after receipt of a study vaccine.
9. Has received systemic immunoglobulins or blood products within 3 months prior to the Screening Visit or plans to receive these during the study.
10. Has donated ≥450 mL of blood products within 28 days prior to the Screening Visit or plans to donate blood products during the study.
11. Plans to participate in an interventional clinical trial of an investigational vaccine or drug while participating in this study.
12. Is an immediate family member or household member of study personnel, study site staff, or Sponsor personnel.

## Exploratory Objective: Detection of SARS-CoV-2 Infection and COVID-19 Cases

The exploratory objective was to conduct an active detection of symptomatic and asymptomatic SARS-CoV-2 infections and COVID-19 cases starting 14 days after study vaccination.

### Derivation of Symptomatic SARS-CoV-2 Infection

For participants with negative pre-booster SARS-CoV-2 status, SARS-CoV-2 infection was considered as either positive status of COVID-19 or asymptomatic SARS-CoV-2 infection, and was defined by either:

- Binding antibody (Ab) levels against SARS-CoV-2 nucleocapsid protein negative at Day 1 that became positive after the investigational product (IP) injection, or
- Positive RT-PCR after the dose of IP.

The date of documented infection was the earlier of:

- Date of positive post-baseline RT-PCR result, or
- Date of positive post-baseline serology test result.

The time to the first SARS-CoV-2 infection was calculated as:

Time to the first SARS-CoV-2 infection = Date of the first documented infection – Date of the IP injection + 1.

Cases were summarized by vaccine group.

### Derivation of Asymptomatic SARS-CoV-2 Infection

In participants with negative pre-booster SARS-CoV-2 status, the incidence of asymptomatic SARS-CoV-2 infection measured by RT-PCR of nasopharyngeal swabs and/or serology tests obtained at post-baseline visits was assessed.

Asymptomatic SARS-CoV-2 infection was identified by absence of symptoms with positive status per RT-PCR or serology tests at a post-baseline visit, specifically:

- Absent of COVID-19 symptoms AND at least 1 from below:
  - Binding Ab levels against SARS-CoV-2 nucleocapsid protein negative at Day 1 that became positive after the investigational product injection, OR
  - Positive RT-PCR test after the investigational product injection.

The date of the asymptomatic infection was the earlier date of positive serology test result based on bAb specific to SARS-CoV-2 nucleocapsid, or positive post-baseline RT-PCR, with absence of symptoms.

The time to the asymptomatic SARS-CoV-2 infection was calculated as:

Time to the asymptomatic SARS-CoV-2 infection = Date of the asymptomatic SARS-CoV-2 infection – Date of the IP injection + 1.

### Derivation of COVID-19

Surveillance for COVID-19 was conducted through monthly contact and scheduled blood collection. For participants reporting COVID-19 symptoms, an illness visit was arranged to collect a nasopharyngeal swab.

Two definitions of the COVID-19 were evaluated:

1. Primary case definition per the Phase 3 study (mRNA-1273-P301): Cases were defined as participants meeting clinical criteria based a positive RT-PCR test result and ≥2 systemic symptoms (fever [≥38ºC], chills, myalgia, headache, sore throat, new olfactory and taste disorder); OR ≥1 respiratory signs/symptoms (cough, shortness of breath or difficulty breathing, or clinical or radiographical evidence of pneumonia) for the COVID-19.
2. Secondary case definition based on CDC criteria: Cases were defined by a positive RT-PCR test on a respiratory sample, and ≥1 systemic or respiratory symptom (fever [≥38ºC] or chills, cough, shortness of breath or difficulty breathing, fatigue, muscle or body aches [not related to exercise], headache, new loss of taste or smell, sore throat, congestion or runny nose, nausea or vomiting, diarrhea).

The date of documented COVID-19 was the later date of either the date of positive RT-PCR test, or the date of eligible symptom(s); the 2 dates were within 14 days of each other.

The time to the first occurrence of COVID-19 was calculated as:

Time to the first occurrence of COVID-19 = Date of documented COVID-19 – Date of the IP injection + 1.

## Immunogenicity Assessments

### Pseudovirus Neutralization Assay

The pseudotyped virus neutralization assay (PsVNA) quantifies nAb using lentivirus particles that express full-length spike proteins on their surface and contain a firefly luciferase reporter gene for quantitative measurements of infection in transduced 293T cells expressing high levels of ACE2 (293T/ACE2 cells) by relative luminescence units (RLU). Serial dilution of antibodies were used to produce a dose−response curve. Neutralization was measured as the serum dilution at which RLU was reduced by 50% (ID_50_) relative to mean RLU in virus control wells (cells + virus but no sample) after subtraction of mean RLU in cell control wells (cells only). Positive controls were included on each assay plate to follow stability over time.

## Definitions of Positive and Negative Pre-Booster SARS-CoV-2 status

Positive SARS-CoV-2 pre-booster status was defined as a positive RT-PCR test for SARS-CoV-2, and/or a positive serology test based on binding antibody (bAb) specific to SARS-CoV-2 nucleocapsid on or before Day 1. Negative SARS-CoV-2 pre-booster status was defined as a negative RT-PCR test for SARS-CoV-2 and a negative serology test based on bAb specific to SARS-CoV-2 nucleocapsid on or before Day 1.

## Definition of Vaccine Seroresponse

Vaccine seroresponse was defined by an increase of SARS-CoV-2 specific neutralizing antibody titer to ≥4 × lower limit of quantification (LLOQ) if the baseline was below <LLOQ, or a ≥4-fold greater rise if pre-booster ≥LLOQ. Two types of baselines for Part A were used to compute the seroresponse:

1. Pre-vaccination (pre-dose 1 of primary series)
2. Pre-booster

Seroresponse based on pre-vaccination was defined as ≥4 × LLOQ for participants with negative SARS-CoV-2 status at pre-vaccination baseline. For this study, the SARS-CoV-2 status of participants at pre-booster baseline was used to impute the SARS-CoV-2 status pre-vaccination baseline. If the participant tested SARS-CoV-2–negative at pre-booster, the serum antibody value of the pre-vaccination baseline was imputed with LLOQ, otherwise, for participants with positive SARS-CoV-2 status at pre-booster, the seroresponse based on pre-vaccination baseline was set to missing.

The seroresponse rate (SRR) at Day 29 was summarized for each vaccine group with its 95% CI calculated with the Clopper-Pearson method. The difference of SRRs at Day 29 for mRNA-1283 and mRNA-1283.211 (each dose level) compared with mRNA-1273 was provided with its 95% CI computed with the Miettinen-Nurminen method.

# Supplementary Figures

## Figure S1. Overview of the study design


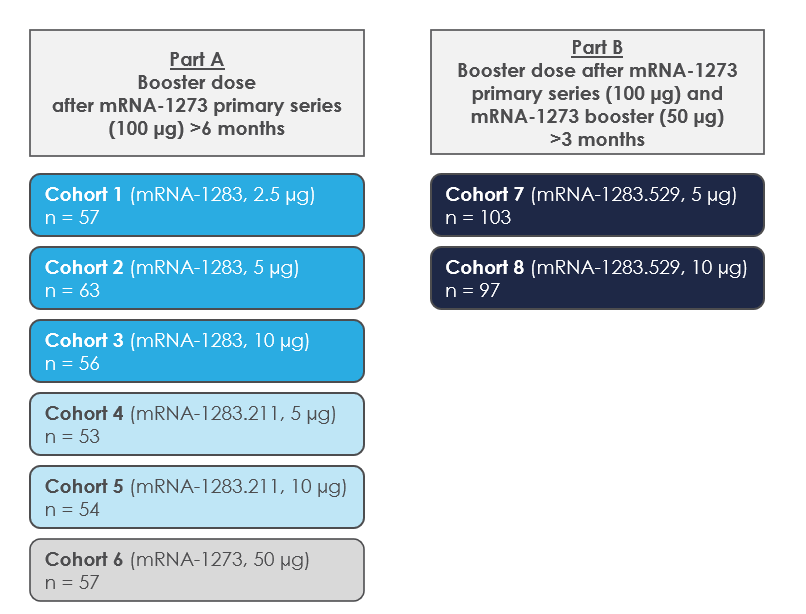


## Figure S2. Participant disposition in Part A (Randomized Set) (A) and Part B (Enrolled Set) (B).


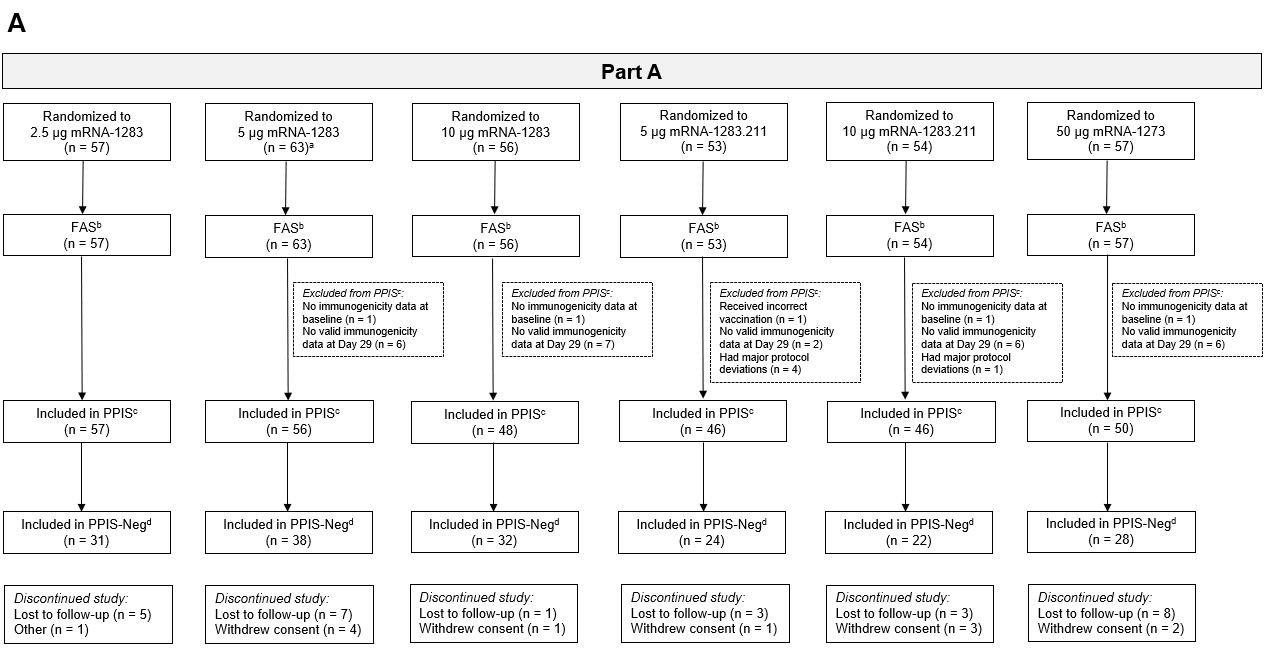


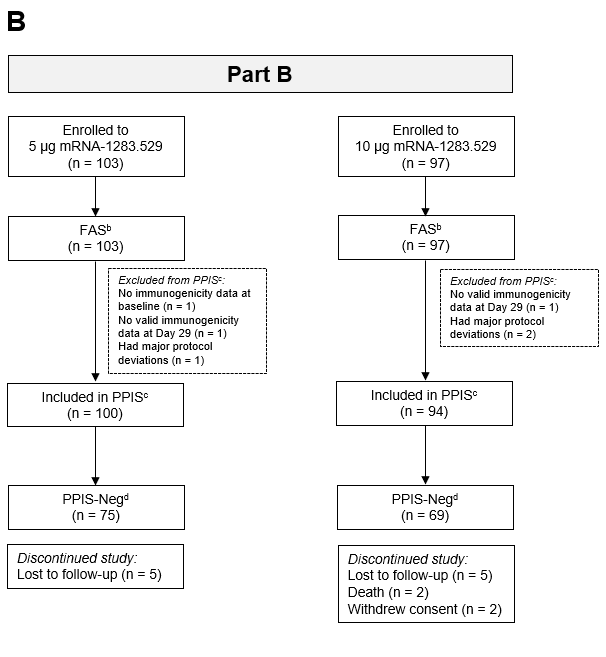


^a^The safety set for the 5-µg mRNA-1283 group comprised 64 participants due to including a participant who was randomized to 5-µg mRNA-1283.211 but received 5-µg mRNA-1283.

^b^The FAS consisted of all randomized (Part A)/enrolled (Part B) participants who received 1 dose of study vaccine.

^c^The PPIS consisted of all participants in the FAS who received the planned study dose, had pre-booster and Day 29 nAb data against prototype SARS-CoV-2, no previous HIV infection, and no major protocol deviations that impacted key or critical data.

^d^The PPIS-Neg (primary set for immunogenicity analysis) consisted of participants in the PPIS who were pre-booster SARS-CoV-2 negative, defined as no virologic or serological evidence of SARS-CoV-2 infection on or before booster, i.e., RT-PCR result was not positive if available pre-booster and a negative bAb specific to SARS-CoV-2 nucleocapsid on or before booster.

*Abbreviations:* bAb, binding antibody; FAS, Full Analysis Set; HIV, human immunodeficiency virus; nAb, neutralizing antibody; PPIS, Per-Protocol Immunogenicity Set; PPIS-Neg, Per-Protocol Immunogenicity Set SARS-CoV-2 negative; RT-PCR, reverse transcription polymerase chain reaction.

# Supplementary Tables

## Table S1. Solicited adverse reactions and grades

| **Reaction** | **Grade 0** | **Grade 1** | **Grade 2** | **Grade 3** | **Grade 4** |
| --- | --- | --- | --- | --- | --- |
| Injection site pain | None | No interference with activity | Repeated use of  over-the-counter pain reliever >24 hours or interferes with activity | Any use of  prescription  pain reliever or  prevents daily  activity | Requires  emergency room  visit or  hospitalization |
| Injection site  erythema (redness) | <25 mm/  <2.5 cm | 25–50 mm/  2.5–5 cm | 51–100 mm/  5.1–10 cm | >100 mm/  >10 cm | Necrosis or  exfoliative  dermatitis |
| Injection site  swelling/induration  (hardness) | <25 mm/  <2.5 cm | 25–50 mm/  2.5–5 cm | 51–100 mm/  5.1–10 cm | >100 mm/  >10 cm | Necrosis |
| Axillary (underarm)  swelling or tenderness  ipsilateral to the  side of injection | None | No interference with activity | Repeated use of  over-the-counter  (non-narcotic) pain  reliever > 24 hours or some interference  with activity | Any use of  prescription  (narcotic) pain  reliever or prevents  daily activity | Requires  emergency room  visit or  hospitalization |
| Headache | None | No interference with activity | Repeated use of  over-the-counter pain reliever > 24 hours or some interference  with activity | Significant; any use of prescription pain reliever or prevents daily activity | Requires  emergency room  visit or  hospitalization |
| Fatigue | None | No interference with activity | Some interference  with activity | Significant; prevents daily activity | Requires  emergency room  visit or  hospitalization |
| Myalgia (muscle  aches all over body) | None | No interference with activity | Some interference  with activity | Significant; prevents daily activity | Requires  emergency room  visit or  hospitalization |
| Arthralgia (joint  aches in several joints) | None | No interference with activity | Some interference  with activity | Significant; prevents daily activity | Requires  emergency room  visit or  hospitalization |
| Nausea/vomiting | None | No interference with activity or 1-2 episodes/24 hours | Some interference  with activity or  >2 episodes/24 hours | Prevents daily  activity, requires  outpatient intravenous hydration | Requires  emergency room  visit or  hospitalization for  hypotensive shock |
| Chills | None | No interference with activity | Some interference  with activity not  requiring medical  intervention | Prevents daily activity and requires  medical intervention | Requires  emergency room  visit or  hospitalization |
| Fever (oral) | <38.0°C  <100.4°F | 38.0–38.4°C  100.4–101.1°F | 38.5–38.9°C  101.2–102.0°F | 39.0–40.0°C  102.1–104.0°F | >40.0°C  >104.0°F |

Events listed above starting >7 days after study vaccine were recorded as AE. Causality for each event was determined per assessment by the investigator.

## Table S2. Number of participants reporting unsolicited TEAEs in Part A (Safety Set^a^)

|  | **mRNA-1283** | | | **mRNA-1283.211** | | **mRNA-1273** |
| --- | --- | --- | --- | --- | --- | --- |
| **n, (%)** | **2.5 µg**  **(N = 57)** | **5 µg**  **(N = 64)** | **10 µg**  **(N = 56)** | **5 µg**  **(N = 52)** | **10 µg**  **(N = 54)** | **50 µg**  **(N = 57)** |
| **Any TEAE** | | | | | | |
| TEAE^b^ | 13 (22.8) | 9 (14.1) | 12 (21.4) | 13 (25.0) | 7 (13.0) | 9 (15.8) |
| SAE^c^ | 2 (3.5) | 1 (1.6) | 2 (3.6) | 3 (5.8) | 3 (5.6) | 2 (3.5) |
| Fatal^c^ | 0 | 0 | 0 | 0 | 0 | 0 |
| MAAE^c^ | 28 (49.1) | 29 (45.3) | 31 (55.4) | 27 (51.9) | 21 (38.9) | 26 (45.6) |
| AESI^c^ | 2 (3.5) | 1 (1.6) | 0 | 0 | 0 | 0 |
| AEs leading to discontinuation^c^ | 0 | 0 | 0 | 0 | 0 | 0 |
| Grade 3/Severe^c^ | 2 (3.5) | 1 (1.6) | 3 (5.4) | 3 (5.8) | 1 (1.9) | 0 |
| **Any vaccine-related TEAE** | | | | | | |
| TEAE^b,d^ | 5 (8.8) | 0 | 1 (1.8) | 2 (3.8) | 2 (3.7) | 1 (1.8) |
| SAE^c^ | 0 | 0 | 0 | 0 | 0 | 0 |
| Fatal^c^ | 0 | 0 | 0 | 0 | 0 | 0 |
| MAAE^c,e^ | 0 | 0 | 0 | 0 | 0 | 1 (1.8) |
| AESI^c^ | 0 | 0 | 0 | 0 | 0 | 0 |
| AEs leading to discontinuation^c,f^ | 0 | 0 | 0 | 0 | 0 | 0 |
| Grade 3/Severe^c^ | 0 | 0 | 0 | 0 | 0 | 0 |

Numbers are based on actual vaccine group and percentages are based on the number of participants in the safety set.

TEAE/AE is defined as any event not present before exposure to study vaccination or any event already present that worsens in intensity or frequency after exposure.

^a^The safety set in Part A comprised all randomized participants who received the study vaccine (N=340); participants were analyzed based on the study vaccine received.

^b^Assessed within 28 days after vaccination.

^c^Assessed throughout the study.

^d^The reported unsolicited TEAEs considered related to study vaccination were as follows: 2.5-µg mRNA-1283, headache, nasal congestion, rhinorrhea, myalgia, chest discomfort, fatigue, injection site paraesthesia, non-cardiac chest pain; 10-µg mRNA-1283, headache; 5-µg mRNA-1283.211, headache, cough, and injection site erythema;
10-µg mRNA-1283.211, headache and injection site pain; 50-µg mRNA-1273, joint swelling.

^e^The reported MAAE considered related to study vaccination included joint swelling (50-µg mRNA-1273). An MAAE was defined as an AE that leads to an unscheduled visit to a health care professional including visits to a study site for unscheduled assessments.

^f^An AESI was defined as an AE (serious or non-serious) of scientific and medical concern specific to the investigational product for which ongoing monitoring and immediate notification by the investigator to the Sponsor is required.

*Abbreviations*: AE, adverse event; AESI, AE of special interest; MAAE, medically attended AE; SAE, serious AE; TEAE, treatment-emergent AE.

## Table S3. Number of participants reporting unsolicited TEAEs in Part B (Safety Set^a^)

|  | **mRNA-1283.529** | |
| --- | --- | --- |
| **n, (%)** | **5 µg**  **(N = 103)** | **10 µg**  **(N = 97)** |
| **Any TEAE** | | |
| TEAE^b^ | 14 (13.6) | 11 (11.3) |
| SAE^c^ | 2 (1.9) | 6 (6.2) |
| Fatal^c^ | 0 | 2 (2.1) |
| MAAE^c^ | 61 (59.2) | 52 (53.6) |
| AESI^c^ | 0 | 3 (3.1) |
| AEs leading to discontinuation^c^ | 0 | 2 (2.1) |
| Grade 3/Severe^c^ | 4 (3.9) | 6 (6.2) |
| **Any vaccine-related TEAE** | | |
| TEAE^b,d^ | 4 (3.9) | 2 (2.1) |
| SAE^c^ | 0 | 0 |
| Fatal^c^ | 0 | 0 |
| MAAE^c,e^ | 2 (1.9) | 0 |
| AESI^c,f^ | 0 | 0 |
| AEs leading to discontinuation^c^ | 0 | 0 |
| Grade 3/Severe^c^ | 1 (1.0) | 0 |

Numbers are based on actual vaccine group and percentages are based on the number of participants in the Safety Set.

TEAE/AE is defined as any event not present before exposure to study vaccination or any event already present that worsens in intensity or frequency after exposure.

^a^The Safety Set in Part B comprised all enrolled participants who received the study vaccine (N=200); the participants were analyzed based on the study vaccine received

^b^Assessed within 28 days after vaccination.

^c^Assessed throughout the study.

^d^Unsolicited TEAEs considered related to study vaccination were as follows: 5-µg mRNA-1283.529, headache, migraine, diarrhea, arthralgia, injection site erythema, injection site pain, and peripheral swelling; 10-µg mRNA-1283.529, vertigo and pain in extremity.

^e^MAAEs considered related to study vaccination were migraine and arthralgia (5-µg mRNA-1283.529). An MAAE was defined as an AE that leads to an unscheduled visit to a health care professional including visits to a study site for unscheduled assessments.

^f^An AESI was defined as an AE (serious or non-serious) of scientific and medical concern specific to the investigational product for which ongoing monitoring and immediate notification by the investigator to the Sponsor is required.

*Abbreviations*: AE, adverse event; AESI, AE of special interest; MAAE, medically attended AE; SAE, serious AE; TEAE, treatment-emergent AE

## Table S4. Summary of neutralizing antibody responses at all time points by SARS-CoV-2 variant in Part A (PPIS-Neg^a^)

| Time Point | **mRNA-1283** | | | **mRNA-1283.211** | | **mRNA-1273** |
| --- | --- | --- | --- | --- | --- | --- |
|  | **2.5 µg**  **(N = 31)** | **5 µg**  **(N = 38)** | **10 µg**  **(N = 32)** | **5 µg**  **(N = 24)** | **10 µg**  **(N = 22)** | **50 µg**  **(N = 28)** |
| **SARS-CoV-2 D614G** | | | | | | |
| **Baseline (Day 1)** | | | | | | |
| n^b^ | 31 | 38 | 32 | 24 | 22 | 28 |
| GMT  (95% CI)^c^ | 177.4  (108.5, 290.0) | 165.4  (114.4, 239.2) | 170.4  (104.4, 278.0) | 143.7  (97.0, 213.1) | 168.6  (74.5, 381.3) | 145.6  (91.9, 230.7) |
| Participants ≥LLOQ, n (%)^d^ | 30 (96.8) | 38 (100) | 32 (100) | 24 (100) | 22 (100) | 27 (96.4) |
| **Day 29** | | | | | | |
| n^b^ | 31 | 38 | 32 | 24 | 22 | 28 |
| GMT  (95% CI)^c^ | 4751.7  (3207.0, 7040.5) | 5666.5  (4149.3, 7738.4) | 7723.0  (5621.7, 10609.7) | 5105.5  (3083.3, 8453.9) | 4729.8  (2572.4, 8696.3) | 3562.9  (2550.1, 4977.9) |
| GMFR  (95% CI)^c^ | 26.8 (16.8, 42.8) | 34.3 (20.9, 56.0) | 45.3 (27.0, 76.0) | 35.5 (18.1, 69.6) | 28.1 (13.2, 59.9) | 24.5 (14.7, 40.7) |
| Seroresponse^e^, n (%)^f^ (95% CI)^g^ | 28 (90.3)  (74.2, 98.0) | 35 (92.1)  (78.6, 98.3) | 30 (93.8)  (79.2, 99.2) | 22 (91.7)  (73.0, 99.0) | 19 (86.4)  (65.1, 97.1) | 26 (92.9)  (76.5, 99.1) |
| **Day 91** | | | | | | |
| n^b^ | 31 | 36 | 32 | 23 | 22 | 28 |
| GMT  (95% CI)^c^ | 3980.4  (2459.2, 6442.6) | 4589.2  (3285.9, 6409.4) | 6754.5  (4604.3, 9909.0) | 4943.1  (3029.0, 8066.9) | 4319.8  (2758.9, 6763.7) | 3155.1  (1980.2, 5027.0) |
| GMFR  (95% CI)^c^ | 22.4 (13.5, 37.4) | 27.1 (16.5, 44.6) | 39.6 (23.0, 68.3) | 34.0 (17.2, 67.1) | 25.6 (12.2, 53.9) | 21.7 (12.4, 38.0) |
| Seroresponse^e^, n (%)^f^ (95% CI)^g^ | 27 (87.1)  (70.2, 96.4) | 32 (88.9)  (73.9, 96.9) | 29 (90.6)  (75.0, 98.0) | 20 (87.0)  (66.4, 97.2) | 19 (86.4)  (65.1, 97.1) | 26 (92.9)  (76.5, 99.1) |
| **Day 181** | | | | | | |
| n^b^ | 30 | 36 | 31 | 24 | 21 | 26 |
| GMT  (95% CI)^c^ | 2476.4  (1400.5, 4378.8) | 2861.4  (1879.2, 4357.1) | 3834.7  (2606.2, 5642.2) | 2960.3  (1805.5, 4853.6) | 2767.2  (1588.2, 4821.6) | 1269.4  (809.8, 1990.0) |
| GMFR  (95% CI)^c^ | 13.9 (7.4, 26.0) | 17.1 (9.9, 29.7) | 23.6 (13.2, 42.1) | 20.6 (11.1, 38.2) | 15.3 (7.6, 30.7) | 8.2 (4.6, 14.6) |
| Seroresponse^e^, n (%)^f^ (95% CI)^g^ | 24 (80.0)  (61.4, 92.3) | 29 (80.6)  (64.0, 91.8) | 27 (87.1)  (70.2, 96.4) | 19 (79.2)  (57.8, 92.9) | 17 (81.0)  (58.1, 94.6) | 17 (65.4)  (44.3, 82.8) |
| **Day 366** | | | | | | |
| n^b^ | 26 | 29 | 30 | 21 | 19 | 22 |
| GMT  (95% CI)^c^ | 1497.4  (723.2, 3100.5) | 3097.6  (2003.1, 4790.2) | 2921.1  (2062.2, 4137.8) | 2013.3  (1225.1, 3308.5) | 1539.6  (772.8, 3067.3) | 1336.1  (739.4, 2414.2) |
| GMFR  (95% CI)^c^ | 9.1 (4.3, 19.2) | 18.0 (9.5, 33.9) | 17.8 (10.3, 30.6) | 13.7 (7.1, 26.4) | 9.8 (4.1, 23.3) | 8.7 (4.3, 17.6) |
| Seroresponse^e^, n (%)^f^ (95% CI)^g^ | 18 (69.2)  (48.2, 85.7) | 25 (86.2)  (68.3, 96.1) | 27 (90.0)  (73.5, 97.9) | 18 (85.7)  (63.7, 97.0) | 12 (63.2)  (38.4 – 83.7) | 14 (63.6)  (40.7, 82.8) |
| **Beta** | | | | | | |
| **Baseline (Day 1)** | | | | | | |
| n^b^ | 30 | 38 | 32 | 24 | 22 | 27 |
| GMT  (95% CI)^c^ | 34.5  (21.0, 56.5) | 36.1  (25.9, 50.3) | 38.8  (24.0, 62.6) | 28.9  (19.0, 44.0) | 29.3  (14.2, 60.4) | 27.9  (17.5, 44.6) |
| Participants ≥LLOQ, n (%)^d^ | 22 (66.7) | 31 (81.6) | 24 (75.0) | 16 (66.7) | 13 (59.1) | 19 (70.4) |
| **Day 29** | | | | | | |
| n^b^ | 30 | 38 | 32 | 24 | 22 | 28 |
| GMT  (95% CI)^c^ | 946.7  (611.4, 1465.7) | 1216.2  (838.9, 1763.3) | 2020.1  (1350.7, 3021.1) | 1566.3  (942.4, 2603.2) | 1224.8  (660.6, 2271.0) | 964.4  (659.6, 1410.1) |
| GMFR  (95% CI)^c^ | 27.4  (16.2, 46.6) | 33.7  (21.1, 53.9) | 52.1  (30.2, 89.8) | 54.2  (27.2, 107.7) | 41.8  (20.8, 84.0) | 35.8  (19.8, 64.6) |
| Seroresponse^e^, n (%)^f^ (95% CI)^g^ | 26 (86.7)  (69.3, 96.2) | 35 (92.1)  (78.6, 98.3) | 31 (96.9)  (83.8, 99.9) | 22 (91.7)  (73.0, 99.0) | 19 (86.4)  (65.1, 97.1) | 25 (92.6)  (75.7, 99.1) |
| **Day 91** | | | | | | |
| n^b^ | 30 | 36 | 32 | 23 | 22 | 28 |
| GMT  (95% CI)^c^ | 794.8  (461.9, 1367.5) | 965.6  (631.0, 1477.7) | 1520.8  (1025.7, 2254.8) | 1105.8  (579.9, 2108.6) | 990.5  (591.0, 1660.0) | 640.2  (384.2, 1066.7) |
| GMFR  (95% CI)^c^ | 23.0  (12.8, 41.6) | 27.0  (16.5, 44.4) | 39.2  (21.9, 70.3) | 38.1  (16.9, 85.9) | 33.8  (17.7, 64.6) | 23.9  (12.7, 45.3) |
| Seroresponse^e^, n (%)^f^ (95% CI)^g^ | 26 (86.7)  (69.3, 96.2) | 33 (91.7)  (77.5, 98.2) | 29 (90.6)  (75.0, 98.0) | 20 (87.0)  (66.4, 97.2) | 20 (90.9)  (70.8, 98.9) | 24 (88.9)  (70.8, 97.6) |
| **Day 181** | | | | | | |
| n^b^ | 30 | 36 | 31 | 24 | 21 | 26 |
| GMT  (95% CI)^c^ | 786.3  (403.1, 1533.7) | 760.2  (438.7, 1317.2) | 1458.4  (916.6, 2320.5) | 967.2  (531.3, 1760.7) | 769.6  (429.6, 1378.9) | 460.5  (266.1, 796.9) |
| GMFR  (95% CI)^c^ | 21.4  (10.1, 45.1) | 21.4  (12.3, 37.2) | 39.3  (21.0, 73.4) | 33.4  (15.8, 70.6) | 24.9  (12.6, 49.4) | 16.5  (8.3, 32.8) |
| Seroresponse^e^, n (%)^f^  (95% CI)^g^ | 23 (79.3)  (60.3, 92.0) | 30 (83.3)  (67.2, 93.6) | 28 (90.3)  (74.2, 98.0) | 22 (91.7)  (73.0, 99.0) | 17 (81.0)  (58.1, 94.6) | 20 (80.0)  (59.3, 93.2) |
| **Day 366** | | | | | | |
| n^b^ | 25 | 29 | 30 | 21 | 19 | 22 |
| GMT  (95% CI)^c^ | 581.2  (279.7, 1207.6) | 1461.8  (855.0, 2499.1) | 1259.6  (789.7, 2009.1) | 790.3  (397.0, 1573.3) | 530.3  (223.4, 1258.4) | 534.2  (252.1, 1132.1) |
| GMFR  (95% CI)^c^ | 17.6  (8.0, 38.7) | 38.2  (20.0, 73.2) | 32.5  (17.6, 59.7) | 27.4  (12.3, 61.0) | 20.0  (7.9, 50.4) | 18.9  (8.0, 44.7) |
| Seroresponse^e^, n (%)^f^  (95% CI)^g^ | 17 (70.8)  (48.9, 87.4) | 27 (93.1)  (77.2, 99.2) | 28 (93.3)  (77.9, 99.2) | 17 (81.0)  (58.1, 94.6) | 14 (73.7)  (48.8, 90.9) | 16 (76.2)  (52.8, 91.8) |
| **Omicron BA.1** | | | | | | |
| **Baseline (Day 1)** | | | | | | |
| n^b^ | 31 | 38 | 32 | 24 | 22 | 28 |
| GMT  (95% CI)^c^ | 30.6  (17.9, 52.2) | 28.3  (19.0, 42.3) | 31.5  (18.9, 52.5) | 22.5  (15.4, 32.9) | 26.0  (13.4, 50.5) | 26.0  (15.0, 44.8) |
| Participants ≥LLOQ, n (%)^d^ | 17 (54.8) | 24 (63.2) | 21 (65.6) | 13 (54.2) | 12 (54.5) | 15 (53.6) |
| **Day 29** | | | | | | |
| n^b^ | 31 | 38 | 32 | 24 | 22 | 28 |
| GMT  (95% CI)^c^ | 1650.4  (935.4, 2911.8) | 1992.7  (1284.6, 3091.3) | 3248.7  (1899.7, 5555.7) | 2748.1  (1571.5, 4805.7) | 1456.0  (682.5, 3106.2) | 1433.8  (898.3, 2288.6) |
| GMFR  (95% CI)^c^ | 54.0  (31.3, 93.0) | 70.3  (39.7, 124.5) | 103.3  (52.1, 204.8) | 122.1  (59.5, 250.6) | 56.1  (24.9, 126.1) | 55.3  (28.7, 106.3) |
| Seroresponse^e^, n (%)^f^ (95% CI)^g^ | 28 (90.3)  (74.2, 98.0) | 35 (92.1)  (78.6, 98.3) | 31 (96.9)  (83.8, 99.9) | 24 (100.0)  (85.8, 100.0) | 19 (86.4)  (65.1, 97.1) | 26 (92.9)  (76.5, 99.1) |
| **Day 91** | | | | | | |
| n^b^ | 31 | 36 | 32 | 23 | 22 | 28 |
| GMT  (95% CI)^c^ | 1104.2  (550.1, 2216.2) | 1291.5  (763.9, 2183.5) | 1878.1  (1164.7, 3028.4) | 1610.3  (727.8, 3562.8) | 1417.7  (778.6, 2581.1) | 812.6  (443.5, 1488.7) |
| GMFR  (95% CI)^c^ | 36.1  (17.8, 73.4) | 44.5  (23.2, 85.6) | 59.7  (31.8, 112.0) | 69.0  (27.9, 170.6) | 54.6  (27.2, 109.7) | 31.3  (16.0, 61.1) |
| Seroresponse^e^, n (%)^f^ (95% CI)^g^ | 26 (83.9)  (66.3, 94.5) | 32 (88.9)  (73.9, 96.9) | 29 (90.6)  (75.0, 98.0) | 19 (82.6)  (61.2, 95.0) | 21 (95.5)  (77.2, 99.9) | 24 (85.7)  (67.3, 96.0) |
| **Day 181** | | | | | | |
| n^b^ | 30 | 36 | 31 | 24 | 21 | 26 |
| GMT  (95% CI)^c^ | 929.6  (444.5, 1944.3) | 999.1  (539.5, 1851.0) | 1605.6  (923.4, 2792.0) | 1279.4  (622.1, 2631.0) | 934.6  (473.5, 1844.9) | 541.5  (279.0, 1050.9) |
| GMFR  (95% CI)^c^ | 29.3  (13.8, 62.3) | 35.5  (17.3, 72.8) | 54.8  (27.5, 109.0) | 56.8  (25.0, 129.3) | 36.0  (17.7, 73.4) | 20.1  (9.6, 41.8) |
| Seroresponse^e^, n (%)^f^ (95% CI)^g^ | 24 (80.0)  (61.4, 92.3) | 29 (80.6)  (64.0, 91.8) | 28 (90.3)  (74.2, 98.0) | 21 (87.5)  (67.6, 97.3) | 18 (85.7)  (63.7, 97.0) | 19 (73.1)  (52.2, 88.4) |
| **Day 366** | | | | | | |
| n^b^ | 26 | 29 | 30 | 21 | 19 | 22 |
| GMT  (95% CI)^c^ | 776.6  (330.5, 1825.0) | 1667.4  (926.6, 3000.3) | 1165.7  (737.9, 1841.6) | 936.3  (419.7, 2089.1) | 618.1  (275.6, 1386.1) | 774.2  (302.5, 1981.7) |
| GMFR  (95% CI)^c^ | 28.8  (11.7, 70.9) | 54.0  (26.9, 108.4) | 38.4  (20.3, 72.5) | 42.5  (16.5, 109.8) | 24.7  (9.9, 61.4) | 27.0  (10.5, 69.5) |
| Seroresponse^e^, n (%)^f^ (95% CI)^g^ | 22 (84.6)  (65.1, 95.6) | 27 (93.1)  (77.2, 99.2) | 28 (93.3)  (77.9, 99.2) | 19 (90.5)  (69.6, 98.8) | 15 (78.9)  (54.4, 93.9) | 17 (77.3)  (54.6, 92.2) |

The LLOQs were 18.5 for the pseudovirus nAb ID_50_ titers against the SARS-CoV-2 D614G, 19.5 for titers against Beta, and 19.85 for titers against Omicron BA.1. Numbers below the LLOQ were replaced by 0.5 × LLOQ.

^a^The PPIS-Neg consisted of participants in the PPIS who were pre-booster SARS-CoV-2 negative, defined as no virologic or serological evidence of SARS-CoV-2 infection on or before booster (ie, RT-PCR result was not positive if available pre-booster and a negative bAb specific to SARS-CoV-2 nucleocapsid on or before booster).

^b^Number of participants with non-missing nAb data at the time point (baseline or post-baseline).

^c^95% CI is calculated based on the t-distribution of the log-transformed values or the difference in the log-transformed values for GMT and GMFR, respectively, then back transformed to the original scale for presentation.

^d^Percentages are based on n.

^e^Pre-booster seroresponse at a participant level is defined as an increase from below the LLOQ to ≥4 × LLOQ if pre-booster baseline nAb titer is <LLOQ, or at least a 4-fold rise if pre-booster baseline nAb titer is ≥LLOQ.

^f^Number of participants meeting the criterion at the time point. Percentage is based on number of participants with non-missing data at baseline and the corresponding post-baseline time point.

^g^95% CI calculated using the Clopper-Pearson method.

*Abbreviations*: bAb, binding antibody; CI, confidence interval; GM, geometric mean; GMFR, geometric mean fold rise; GMT, geometric mean titer; LLOQ, lower limit of quantification; mRNA, messenger RNA; nAb, neutralizing antibody; PPIS, Per-Protocol Immunogenicity Set; PPIS-Neg, Per-Protocol Immunogenicity Set SARS-CoV-2–negative; RT-PCR, reverse transcription-polymerase chain reaction.

## Table S5. Geometric mean ratios of neutralizing antibody GMTs of mRNA-1283 and mRNA-1283.211 versus mRNA-1273 by SARS-CoV-2 variant in Part A (PPIS-Neg^a^)

| Time Point | **mRNA-1283** | | | **mRNA-1283.211** | | **mRNA-1273** |
| --- | --- | --- | --- | --- | --- | --- |
|  | **2.5 µg**  **(N = 31)** | **5 µg**  **(N = 38)** | **10 µg**  **(N = 32)** | **5 µg**  **(N = 24)** | **10 µg**  **(N = 22)** | **50 µg**  **(N = 28)** |
| **SARS-CoV-2 D614G** | | | | | | |
| **Day 29** | | | | | | |
| n^b^ | 31 | 38 | 32 | 24 | 22 | 28 |
| GMR^c^  (95% CI) | 1.334  (0.779, 2.283) | 1.590  (0.952, 2.657) | 2.168  (1.272, 3.695) | 1.433  (0.808, 2.542) | 1.328  (0.738, 2.388) | – |
| **Day 91** | | | | | | |
| n^b^ | 31 | 36 | 32 | 23 | 22 | 28 |
| GMR^c^  (95% CI) | 1.262  (0.707, 2.250) | 1.455  (0.832, 2.544) | 2.141  (1.206, 3.801) | 1.567  (0.839, 2.925) | 1.369  (0.728, 2.576) | – |
| **Day 181** | | | | | | |
| n^b^ | 30 | 36 | 31 | 24 | 21 | 26 |
| GMR^c^  (95% CI) | 1.951  (1.017, 3.742) | 2.254  (1.206, 4.214) | 3.021  (1.583, 5.765) | 2.332  (1.172, 4.640) | 2.180  (1.068, 4.448) | – |
| **Day 366** | | | | | | |
| n^b^ | 26 | 29 | 30 | 21 | 19 | 22 |
| GMR^c^  (95% CI) | 1.121  (0.529, 2.375) | 2.318  (1.114, 4.826) | 2.186  (1.056, 4.527) | 1.507  (0.683, 3.324) | 1.152  (0.512, 2.596) | – |
| **Beta** | | | | | | |
| **Day 29** | | | | | | |
| n^b^ | 30 | 38 | 32 | 24 | 22 | 28 |
| GMR^c^  (95% CI) | 0.947  (0.515–1.740) | 1.216  (0.682–2.167) | 2.020  (1.109–3.680) | 1.566  (0.822–2.982) | 1.225  (0.633–2.368) | – |
| **Day 91** | | | | | | |
| n^b^ | 30 | 36 | 32 | 23 | 22 | 28 |
| GMR^c^  (95% CI) | 1.188  (0.601–2.348) | 1.443  (0.750–2.776) | 2.273  (1.161–4.448) | 1.653  (0.797–3.426) | 1.480  (0.708–3.096) | – |
| **Day 181** | | | | | | |
| n^b^ | 30 | 36 | 31 | 24 | 21 | 26 |
| GMR^c^  (95% CI) | 1.603  (0.716–3.587) | 1.582  (0.734–3.411) | 3.034  (1.372–6.709) | 2.012  (0.866–4.678) | 1.601  (0.668–3.836) | – |
| **Day 366** | | | | | | |
| n^b^ | 25 | 29 | 30 | 21 | 19 | 22 |
| GMR^c^  (95% CI) | 0.961  (0.383–2.407) | 2.489  (1.032–6.006) | 2.145  (0.895–5.143) | 1.346  (0.521–3.475) | 0.903  (0.341–2.390) | – |
| **Omicron BA.1** | | | | | | |
| **Day 29** | | | | | | |
| n^b^ | 31 | 38 | 32 | 24 | 22 | 28 |
| GMR^c^  (95% CI) | 1.151  (0.550, 2.410) | 1.390  (0.686, 2.815) | 2.266  (1.088, 4.717) | 1.917  (0.871, 4.216) | 1.015  (0.453, 2.277) | – |
| **Day 91** | | | | | | |
| n^b^ | 31 | 36 | 32 | 23 | 22 | 28 |
| GMR^c^  (95% CI) | 1.359  (0.603, 3.064) | 1.589  (0.724, 3.487) | 2.311  (1.031, 5.180) | 1.982  (0.824, 4.766) | 1.745  (0.718, 4.242) | – |
| **Day 181** | | | | | | |
| n^b^ | 30 | 36 | 31 | 24 | 21 | 26 |
| GMR^c^  (95% CI) | 1.717  (0.695, 4.238) | 1.845  (0.775, 4.395) | 2.965  (1.209, 7.271) | 2.363  (0.909, 6.138) | 1.726  (0.642, 4.643) | – |
| **Day 366** | | | | | | |
| n^b^ | 26 | 29 | 30 | 21 | 19 | 22 |
| GMR^c^  (95% CI) | 1.003  (0.368, 2.734) | 2.154  (0.809, 5.730) | 1.506  (0.570, 3.978) | 1.209  (0.421, 3.477) | 0.798  (0.270, 2.360) | – |

Antibody values reported as below the LLOQ were replaced by 0.5 × LLOQ.

The log-transformed antibody titers were analyzed using an ANCOVA model with the treatment variable as fixed effect, adjusting for age group (18-55, ≥56 years) and the pre-booster baseline SARS-CoV-2 infection status. Coefficients for LS means use margins by level. In PPIS-Neg, pre-booster infection status was not a covariate. The resulted LS means, difference of LS means, and 95% CI are back transformed to the original scale for presentation.

^a^The PPIS-Neg consisted of participants in the PPIS who were pre booster SARS-CoV-2 negative, defined as no virologic or serological evidence of SARS-CoV-2 infection on or before booster, ie, RT-PCR result was not positive if available pre-booster and a negative bAb specific to SARS-CoV-2 nucleocapsid on or before booster.

^b^Number of participants with non-missing data at the corresponding time point.

^c^GMR of mRNA-1283 and mRNA-1283.211 versus mRNA-1273.

*Abbreviations*: ANCOVA, analysis of covariance; bAb, binding antibody; CI, confidence interval; GMR, geometric mean ratio; GMT, geometric mean titer; LLOQ, lower limit of quantification; LS, least squares; PPIS, Per-Protocol Immunogenicity Set; PPIS-Neg, Per-Protocol Immunogenicity Set SARS-CoV-2–negative; RT-PCR, reverse transcription-polymerase chain reaction.

## Table S6. Summary of neutralizing antibody responses at all time points by SARS-CoV-2 variant in Part A (PPIS^a^) – sensitivity analysis

| Time Point | **mRNA-1283** | | | **mRNA-1283.211** | | **mRNA-1273** |
| --- | --- | --- | --- | --- | --- | --- |
|  | **2.5 µg**  **(N = 57)** | **5 µg**  **(N = 56)** | **10 µg**  **(N = 48)** | **5 µg**  **(N = 46)** | **10 µg**  **(N = 46)** | **50 µg**  **(N = 50)** |
| **SARS-CoV-2 D614G** | | | | | | |
| **Baseline (Day 1)** | | | | | | |
| n^b^ | 57 | 56 | 48 | 46 | 46 | 50 |
| GMT  (95% CI)^c^ | 517.3  (317.3, 843.4) | 311.0  (195.0, 496.2) | 340.3  (201.6, 574.4) | 438.4  (263.5, 729.6) | 600.2  (317.7, 1134.0) | 395.6  (238.9, 655.1) |
| Participants ≥LLOQ, n (%)^d^ | 56 (98.2) | 56 (100) | 48 (100) | 46 (100) | 46 (100) | 49 (98.0) |
| **Day 29** | | | | | | |
| n^b^ | 57 | 56 | 48 | 46 | 46 | 50 |
| GMT  (95% CI)^c^ | 5357.1  (4057.5, 7073.0) | 5608.1  (4334.7, 7255.5) | 7104.8  (5237.7, 9637.6) | 5363.1  (3542.0, 8120.5) | 5325.9  (3657.7, 7755.0) | 4608.9  (3314.7, 6408.4) |
| GMFR  (95% CI)^c^ | 10.4  (6.5, 16.6) | 18.0  (11.1, 29.2) | 20.9  (12.4, 35.1) | 12.2  (7.3, 20.5) | 8.9  (5.2, 15.1) | 11.6  (7.6, 17.8) |
| Seroresponse^e^, n (%)^f^ (95% CI)^g^ | 35 (61.4)  (47.6, 74.0) | 44 (78.6)  (65.6, 88.4) | 36 (75.0)  (60.4, 86.4) | 31 (67.4)  (52.0, 80.5) | 24 (52.2)  (36.9, 67.1) | 39 (78.0)  (64.0, 88.5) |
| **Day 91** | | | | | | |
| n^b^ | 56 | 54 | 48 | 45 | 45 | 49 |
| GMT  (95% CI)^c^ | 3919.4  (2866.9, 5358.4) | 4375.7  (3346.4, 5721.7) | 5941.4  (4288.8, 8230.9) | 3940.6  (2691.9, 5768.5) | 3947.7  (2820.3, 5525.6) | 3107.9  (2175.8, 4439.2) |
| GMFR  (95% CI)^c^ | 7.7  (4.6, 12.9) | 13.5  (8.1, 22.8) | 17.5  (9.9, 30.9) | 8.7  (4.9, 15.4) | 7.1  (4.0, 12.6) | 8.5  (5.0, 14.3) |
| Seroresponse^e^, n (%)^f^ (95% CI)^g^ | 32 (57.1)  (43.2, 70.3) | 40 (74.1)  (60.3, 85.0) | 33 (68.8)  (53.7, 81.3) | 26 (57.8)  (42.2, 72.3) | 23 (51.1)  (35.8, 66.3) | 36 (73.5)  (58.9, 85.1) |
| **Day 181** | | | | | | |
| n^b^ | 56 | 51 | 47 | 45 | 45 | 46 |
| GMT  (95% CI)^c^ | 2251.9  (1595.2, 3179.0) | 2742.2  (1952.3, 3851.7) | 3082.6  (2283.1, 4162.0) | 2567.1  (1792.8, 3675.7) | 2277.8  (1621.3, 3200.0) | 1521.0  (1043.5, 2217.0) |
| GMFR  (95% CI)^c^ | 4.3  (2.4, 7.4) | 9.8  (5.7, 16.7) | 9.2  (5.0, 16.8) | 5.9  (3.5, 10.2) | 3.6  (2.0, 6.4) | 3.9  (2.4, 6.3) |
| Seroresponse^e^, n (%)^f^ (95% CI)^g^ | 28 (50.0)  (36.3, 63.7) | 37 (72.5)  (58.3, 84.1) | 30 (63.8)  (48.5, 77.3) | 21 (46.7)  (31.7, 62.1) | 19 (42.2)  (27.7, 57.8) | 21 (45.7)  (30.9, 61.0) |
| **Day 366** | | | | | | |
| n^b^ | 49 | 42 | 46 | 39 | 42 | 42 |
| GMT  (95% CI)^c^ | 1469.6  (982.9, 2197.3) | 2432.2  (1702.3, 3475.1) | 2503.4  (1897.2, 3303.4) | 1644.0  (1143.4, 2363.8) | 1395.1  (952.5, 2043.3) | 1268.9  (861.7, 1868.5) |
| GMFR  (95% CI)^c^ | 2.7  (1.5, 5.0) | 8.7  (4.7, 16.0) | 7.3  (4.2, 12.8) | 3.9  (2.1, 7.0) | 2.2  (1.2, 4.3) | 3.0  (1.7, 5.2) |
| Seroresponse^e^, n (%)^f^ (95% CI)^g^ | 20 (40.8)  (27.0, 55.8) | 29 (69.0)  (52.9, 82.4) | 30 (65.2)  (49.8, 78.6) | 19 (48.7)  (32.4, 65.2) | 14 (33.3)  (19.6, 49.5) | 16 (38.1)  (23.6, 54.4) |
| **Beta** | | | | | | |
| **Baseline (Day 1)** | | | | | | |
| n^b^ | 56 | 56 | 48 | 45 | 46 | 49 |
| GMT  (95% CI)^c^ | 119.8  (71.4, 201.1) | 71.8  (44.0, 117.3) | 84.9  (49.5, 145.6) | 90.6  (55.3, 148.3) | 119.5  (60.9, 234.5) | 77.6  (46.0, 131.0) |
| Participants ≥LLOQ, n (%)^d^ | 45 (80.4) | 46 (82.1) | 39 (81.3) | 37 (82.2) | 36 (78.3) | 40 (81.6) |
| **Day 29** | | | | | | |
| n^b^ | 56 | 56 | 48 | 45 | 46 | 50 |
| GMT  (95% CI)^c^ | 1233.7  (913.5, 1666.2) | 1325.4  (972.3, 1806.7) | 1810.8  (1290.4, 2541.2) | 1429.9  (997.1, 2050.5) | 1444.9  (970.4, 2151.6) | 1167.2  (788.7, 1727.5) |
| GMFR  (95% CI)^c^ | 10.3  (6.2, 17.0) | 18.5  (11.6, 29.5) | 21.3  (12.2, 37.1) | 15.8  (9.0, 27.7) | 12.1  (7.1, 20.6) | 15.4  (9.7, 24.4) |
| Seroresponse^e^, n (%)^f^ (95% CI)^g^ | 33 (58.9)  (45.0, 71.9) | 44 (78.6)  (65.6, 88.4) | 35 (72.9)  (58.2, 84.7) | 31 (68.9)  (53.4, 81.8) | 28 (60.9)  (45.4, 74.9) | 36 (73.5)  (58.9, 85.1) |
| **Day 91** | | | | | | |
| n^b^ | 55 | 54 | 48 | 44 | 45 | 49 |
| GMT  (95% CI)^c^ | 839.1  (596.6, 1180.0) | 943.1  (670.2, 1327.1) | 1290.8  (947.4, 1758.5) | 924.5  (612.3, 1395.9) | 896.5  (610.5, 1316.4) | 660.9  (435.6, 1002.6) |
| GMFR  (95% CI)^c^ | 7.2  (4.0, 12.9) | 12.9  (7.6, 21.7) | 15.2  (8.4, 27.7) | 9.9  (5.3, 18.6) | 8.0  (4.5, 14.3) | 9.2  (5.4, 15.7) |
| Seroresponse^e^, n (%)^f^ (95% CI)^g^ | 32 (58.2)  (44.1, 71.3) | 41 (75.9)  (62.4, 86.5) | 32 (66.7)  (51.6, 79.6) | 26 (59.1)  (43.2, 73.7) | 25 (55.6)  (40.0, 70.4) | 34 (70.8)  (55.9, 83.0) |
| **Day 181** | | | | | | |
| n^b^ | 55 | 51 | 47 | 45 | 45 | 46 |
| GMT  (95% CI)^c^ | 735.9  (497.4, 1089.0) | 812.4  (520.1, 1269.1) | 1240.3  (890.3, 1728.0) | 843.2  (573.0, 1240.7) | 656.7  (450.7, 957.0) | 560.5  (368.4, 852.9) |
| GMFR  (95% CI)^c^ | 6.1  (3.2, 11.5) | 12.9  (7.7, 21.7) | 14.8  (7.9, 27.6) | 9.2  (5.0, 17.0) | 5.2  (2.8, 9.6) | 7.3  (4.3, 12.5) |
| Seroresponse^e^, n (%)^f^ (95% CI)^g^ | 27 (50.0)  (36.1, 63.9) | 28 (74.5)  (60.4, 85.7) | 32 (68.1)  (52.9, 80.9) | 27 (61.4)  (45.5, 75.6) | 21 (46.7)  (31.7, 62.1) | 26 (57.8)  (42.2, 72.3) |
| **Day 366** | | | | | | |
| n^b^ | 48 | 42 | 46 | 39 | 42 | 42 |
| GMT  (95% CI)^c^ | 531.2  (346.1, 815.2) | 1005.8  (637.6, 1586.5) | 1017.7  (721.4, 1435.7) | 650.7  (428.5, 988.1) | 485.1  (301.4, 780.7) | 481.6  (301.8, 768.5) |
| GMFR  (95% CI)^c^ | 4.0  (2.0, 8.1) | 15.3  (7.7, 30.4) | 11.6  (6.2, 21.7) | 7.2  (3.7, 14.2) | 4.1  (2.0, 8.3) | 5.6  (3.0, 10.8) |
| Seroresponse^e^, n (%)^f^ (95% CI)^g^ | 21 (44.7)  (30.2, 59.9) | 31 (73.8)  (58.0, 86.1) | 33 (71.7)  (56.5, 84.0) | 18 (47.4)  (31.0, 64.2) | 18 (42.9)  (27.7, 59.0) | 22 (53.7)  (37.4, 69.3) |
| **Omicron BA.1** | | | | | | |
| **Baseline (Day 1)** | | | | | | |
| n^b^ | 57 | 56 | 48 | 46 | 46 | 50 |
| GMT  (95% CI)^c^ | 127.4 (70.4, 230.6) | 62.7 (35.7, 110.3) | 85.8 (45.6, 161.6) | 81.7 (47.3, 141.0) | 129.3 (63.6, 262.7) | 72.3 (40.0, 130.7) |
| Participants ≥LLOQ, n (%)^d^ | 42 (73.7) | 40 (71.4) | 37 (77.1) | 35 (76.1) | 36 (78.3) | 35 (70.0) |
| **Day 29** | | | | | | |
| n^b^ | 56 | 56 | 48 | 45 | 46 | 50 |
| GMT  (95% CI)^c^ | 2090.2 (1425.8, 3064.2) | 2349.7 (1586.2, 3480.7) | 3066.6 (1940.8, 4845.4) | 2827.3 (1891.9, 4225.1) | 2196.6 (1368.2, 3526.6) | 1682.6 (1083.7, 2612.5 |
| GMFR  (95% CI)^c^ | 16.4 (9.3, 29.0) | 37.4 (22.3, 62.9) | 35.7 (18.5, 69.0) | 34.6 (19.1, 62.9) | 17.0 (9.6, 30.0) | 23.3 (13.8, 39.2) |
| Seroresponse^e^, n (%)^f^ (95% CI)^g^ | 36 (63.2)  (49.3, 75.6) | 45 (80.4)  (67.6, 89.8) | 38 (81.3)  (67.4, 91.1) | 39 (84.8)  (71.1, 93.7) | 34 (73.9)  (58.9, 85.7) | 40 (80.0)  (66.3, 90.0) |
| **Day 91** | | | | | | |
| n^b^ | 56 | 54 | 48 | 45 | 45 | 49 |
| GMT  (95% CI)^c^ | 1306.1 (859.8, 1984.1) | 1365.1 (886.4, 2102.4) | 1867.2 (1280.5, 2722.6) | 1592.1 (979.8, 2587.0) | 1576.8 (1008.8, 2464.4) | 838.4 (512.7, 1371.0) |
| GMFR  (95% CI)^c^ | 10.6  (5.6, 20.0) | 20.8  (11.4, 37.8) | 21.8  (11.6, 40.9) | 18.6  (9.7, 35.7) | 13.0  (7.1, 23.7) | 12.0  (6.8, 21.3) |
| Seroresponse^e^, n (%)^f^ (95% CI)^g^ | 32 (57.1)  (43.2, 70.3) | 41 (75.9)  (62.4, 86.5) | 33 (68.8)  (53.7, 81.3) | 30 (66.7)  (51.0, 80.0) | 29 (64.4)  (48.8, 78.1) | 33 (67.3)  (52.5, 80.1) |
| **Day 181** | | | | | | |
| n^b^ | 56 | 51 | 47 | 45 | 44 | 46 |
| GMT  (95% CI)^c^ | 932.6 (613.6, 1417.3) | 1025.5 (621.8, 1691.0) | 1433.9 (960.8, 2139.9) | 1059.5 (687.6, 1632.7) | 997.8 (663.9, 1499.5) | 711.0 (432.2, 1169.7) |
| GMFR  (95% CI)^c^ | 7.0 (3.5, 14.0) | 18.4 (9.6, 35.1) | 17.1 (8.4, 34.8) | 13.3 (6.8, 26.2) | 8.0 (4.2, 15.3) | 9.6 (5.5, 17.0) |
| Seroresponse^e^, n (%)^f^ (95% CI)^g^ | 29 (51.8)  (38.0, 65.3) | 36 (70.6)  (56.2, 82.5) | 33 (70.2)  (55.1, 82.7) | 27 (60.0)  (44.3, 74.3) | 24 (54.5)  (38.8, 69.6) | 26 (56.5)  (41.4, 71.1) |
| **Day 366** | | | | | | |
| n^b^ | 49 | 42 | 46 | 39 | 42 | 42 |
| GMT  (95% CI)^c^ | 747.4 (466.0, 1198.7) | 1279.9 (779.2, 2102.2) | 1202.6 (853.5, 1694.5) | 798.0 (499.9, 1273.9) | 766.7 (470.2, 1250.3) | 681.1 (374.1, 1240.0) |
| GMFR  (95% CI)^c^ | 5.7 (2.7, 12.3) | 21.8 (10.6, 44.7) | 13.7 (7.0, 26.9) | 9.9 (4.6, 21.1) | 5.6 (2.8, 11.3) | 8.1 (4.1, 16.1) |
| Seroresponse^e^, n (%)^f^ (95% CI)^g^ | 26 (53.1)  (38.3, 67.5) | 34 (81.0)  (65.9, 91.4) | 34 (73.9)  (58.9, 85.7) | 24 (61.5)  (44.6, 76.6) | 21 (50.0)  (34.2, 65.8) | 24 (57.1)  (41.0, 72.3) |

The LLOQ was 18.5 for the pseudovirus neutralizing antibody ID_50_ titers against SARS-CoV-2 D614G, 19.5 for titers against Beta , and 19.85 for titers against Omicron BA.1. Numbers below the LLOQ were replaced by 0.5 × LLOQ.

^a^The PPIS consisted of all randomized participants who received the planned study vaccine, had pre-booster and Day 29 nAb against prototype virus strain, no previous HIV infection, and no major protocol deviations that impacted key or critical data.

^b^Number of participants with non-missing nAb data at the time point (baseline or post-baseline).

^c^95% CI is calculated based on the t-distribution of the log-transformed values or the difference in the log-transformed values for GMT and GMFR, respectively, then back transformed to the original scale for presentation.

^d^Percentages are based on n.

^e^Pre-booster seroresponse at a participant level is defined as an increase from below the LLOQ to ≥4 × LLOQ if pre-booster baseline nAb titer is <LLOQ, or at least a 4-fold rise if pre-booster baseline nAb titer is ≥LLOQ.

^f^Number of participants meeting the criterion at the time point. Percentage is based on number of participants with non-missing data at baseline and the corresponding post-baseline time point.

^g^95% CI is calculated using the Clopper-Pearson method.

*Abbreviations*: CI, confidence interval; GM, geometric mean; GMFR, geometric mean fold rise; GMT, geometric mean titer; HIV, human immunodeficiency virus; LLOQ, lower limit of quantification; mRNA, messenger RNA; nAb, neutralizing antibody; PPIS, Per-Protocol Immunogenicity Set.

## Table S7. Geometric mean ratios of neutralizing antibody GMTs of mRNA-1283 and mRNA-1283.211 versus mRNA-1273 by SARS-CoV-2 variant in Part A (PPIS^a^) – sensitivity analysis

| Time Point | **mRNA-1283** | | | **mRNA-1283.211** | | **mRNA-1273** |
| --- | --- | --- | --- | --- | --- | --- |
|  | **2.5 µg**  **(N = 57)** | **5 µg**  **(N = 56)** | **10 µg**  **(N = 48)** | **5 µg**  **(N = 46)** | **10 µg**  **(N = 46)** | **50 µg**  **(N = 50)** |
| **SARS-CoV-2 D614G** | | | | | | |
| **Day 29** | | | | | | |
| n^b^ | 57 | 56 | 48 | 46 | 46 | 50 |
| GMR^c^  (95% CI) | 1.144  (0.735, 1.780) | 1.192  (0.763, 1.862) | 1.517  (0.957, 2.406) | 1.145  (0.719, 1.825) | 1.137  (0.714, 1.812) | – |
| **Day 91** | | | | | | |
| n^b^ | 56 | 54 | 48 | 45 | 45 | 49 |
| GMR^c^  (95% CI) | 1.248  (0.796, 1.956) | 1.411  (0.895, 2.225) | 1.891  (1.186, 3.016) | 1.254  (0.781, 2.015) | 1.257  (0.782, 2.019) | – |
| **Day 181** | | | | | | |
| n^b^ | 56 | 51 | 47 | 45 | 45 | 46 |
| GMR^c^  (95% CI) | 1.535  (0.959, 2.459) | 1.894  (1.168, 3.071) | 2.102  (1.287, 3.432) | 1.750  (1.066, 2.873) | 1.553  (0.946, 2.549) | – |
| **Day 366** | | | | | | |
| n^b^ | 49 | 42 | 46 | 39 | 42 | 42 |
| GMR^c^  (95% CI) | 1.135  (0.689, 1.869) | 1.878  (1.120, 3.150) | 1.933  (1.165, 3.207) | 1.269  (0.750, 2.150) | 1.077  (0.642, 1.807) | – |
| **Beta** | | | | | | |
| **Day 29** | | | | | | |
| n^b^ | 56 | 56 | 48 | 45 | 46 | 50 |
| GMR^c^  (95% CI) | 0.990 (0.614, 1.596) | 1.073 (0.644, 1.733) | 1.453 (0.885, 2.385) | 1.147 (0.693, 1.898) | 1.159 (0.703, 1.913) | – |
| **Day 91** | | | | | | |
| n^b^ | 55 | 54 | 48 | 44 | 45 | 49 |
| GMR^c^  (95% CI) | 1.190 (0.718, 1.973) | 1.354 (0.813, 2.254) | 1.831 (1.087, 3.086) | 1.312 (0.769, 2.236) | 1.272 (0.748, 2.161) | – |
| **Day 181** | | | | | | |
| n^b^ | 55 | 51 | 47 | 45 | 45 | 46 |
| GMR^c^  (95% CI) | 1.255 (0.722, 2.183) | 1.438 (0.819, 2.526) | 2.141 (1.209, 3.792) | 1.416 (0.793, 2.531) | 1.134 (0.637, 2.020) | – |
| **Day 366** | | | | | | |
| n^b^ | 48 | 42 | 46 | 39 | 42 | 42 |
| GMR^c^  (95% CI) | 0.976 (0.539, 1.767) | 1.880 (1.023, 3.458) | 1.903 (1.048, 3.454) | 1.204 (0.645, 2.248) | 0.907 (0.493. 1.668) | – |
| **Omicron BA.1** | | | | | | |
| **Day 29** | | | | | | |
| n^b^ | 57 | 56 | 48 | 46 | 46 | 50 |
| GMR^c^  (95% CI) | 1.179 (0.664, 2.095) | 1.342 (0.752, 2.396) | 1.730 (0.951, 3.148) | 1.595 (0.871, 2.921) | 1.239 (0.677, 2.270) | – |
| **Day 91** | | | | | | |
| n^b^ | 56 | 54 | 48 | 45 | 45 | 49 |
| GMR^c^  (95% CI) | 1.453 (0.796, 2.654) | 1.547 (0.840, 2.847) | 2.078 (1.112, 3.881) | 1.772 (0.939, 3.343) | 1.755 (0.930, 3.311) | – |
| **Day 181** | | | | | | |
| n^b^ | 56 | 51 | 47 | 45 | 44 | 46 |
| GMR^c^  (95% CI) | 1.329 (0.721, 2.452) | 1.512 (0.807, 2.833) | 2.044 (1.081, 3.866) | 1.510 (0.793, 2.877) | 1.422 (0.744, 2.719) | – |
| **Day 366** | | | | | | |
| n^b^ | 49 | 42 | 46 | 39 | 42 | 42 |
| GMR^c^  (95% CI) | 1.079 (0.559, 2.083) | 1.847 (0.934, 3.654) | 1.736 (0.890, 3.383) | 1.152 (0.575, 2.307) | 1.107 (0.559. 2.189) | – |

Antibody values reported as below the LLOQ were replaced by 0.5 × LLOQ.

The log-transformed antibody titers were analyzed using an ANCOVA model with the treatment variable as fixed effect, adjusting for age group (18-55, ≥56 years) and the pre-booster baseline SARS-CoV-2 infection status. Coefficients for LS means use margins by level. The resulted LS means, difference of LS means, and 95% CI are back transformed to the original scale for presentation.

^a^The PPIS consisted of all randomized participants who received the planned study vaccine, had pre-booster and Day 29 nAb data against prototype virus strain, no previous HIV infection, and no major protocol deviations that impacted key or critical data.

^b^Number of participants with non-missing data at the corresponding time point.

^c^GMR of mRNA-1283 and mRNA-1283.211 versus mRNA-1273.

*Abbreviations*: ANCOVA, analysis of covariance; CI, confidence interval; GMR, geometric mean ratio; GMT, geometric mean titer; HIV, human immunodeficiency virus; LLOQ, lower limit of quantification; LS, least squares; nAb, neutralizing antibody; PPIS, Per-Protocol Immunogenicity Set.

## Table S8. Summary of neutralizing antibody responses at all time points by SARS-CoV-2 variant in Part B (PPIS-Neg^a^)

| Time Point | **mRNA-1283.529** | |
| --- | --- | --- |
|  | **5 µg**  **(N = 75)** | **10 µg**  **(N = 69)** |
| **SARS-CoV-2 D614G** | | |
| **Baseline (Day 1)** | | |
| n^b^ | 75 | 69 |
| GMT  (95% CI)^c^ | 1168.5 (898.7, 1519.2) | 1793.9 (1394.3, 2308.1) |
| Participants ≥LLOQ, n (%)^d^ | 75 (100) | 69 (100) |
| **Day 29** | | |
| n^b^ | 75 | 69 |
| GMT  (95% CI)^c^ | 2979.8  (2376.9, 3735.7) | 4841.4 (3739.7, 6267.7) |
| GMFR  (95% CI)^c^ | 2.6 (2.1, 3.0) | 2.7 (2.2, 3.3) |
| Seroresponse^e^, n (%)^f^ (95% CI)^g^ | 14 (18.7) (10.6, 29.3) | 16 (23.2) (13.9, 34.9) |
| **Day 91** | | |
| n^b^ | 73 | 66 |
| GMT  (95% CI)^c^ | 2021.4 (1531.3, 2668.2) | 3243.7 (2519.5, 4176.0) |
| GMFR  (95% CI)^c^ | 1.7 (1.4, 2.2) | 1.8 (1.5, 2.2) |
| Seroresponse^e^, n (%)^f^ (95% CI)^g^ | 10 (13.7)  (6.8, 23.8) | 9 (13.6) (6.4, 24.3) |
| **Day 181** | | |
| n^b^ | 73 | 65 |
| GMT  (95% CI)^c^ | 1585.7 (1164.4, 2159.3) | 2510.2 (1821.9, 3458.7) |
| GMFR  (95% CI)^c^ | 1.4 (1.0, 1.9) | 1.4 (1.0, 1.9) |
| Seroresponse^e^, n (%)^f^ (95% CI)^g^ | 17 (23.3) (14.2, 34.6) | 13 (20.0) (11.1, 31.8) |
| **Day 366** | | |
| n^b^ | 43 | 39 |
| GMT  (95% CI)^c^ | 1274.1 (802.1, 2023.9) | 2341.8  (1490.1, 3680.4) |
| GMFR  (95% CI)^c^ | 1.0 (0.6, 1.5) | 1.4 (0.8, 2.3) |
| Seroresponse^e^, n (%)^f^ (95% CI)^g^ | 8 (18.6) (8.4, 33.4) | 11 (28.2) (15.0, 44.9) |
| **Omicron BA.1** | | |
| **Baseline (Day 1)** | | |
| n^b^ | 75 | 69 |
| GMT  (95% CI)^c^ | 217.6  (162.0, 292.4) | 349.2  (239.7, 508.7) |
| Participants ≥LLOQ, n (%)^d^ | 72 (96.0) | 69 (100) |
| **Day 29** | | |
| n^b^ | 75 | 69 |
| GMT  (95% CI)^c^ | 1562.9  (1113.4, 2194.0) | 2723.0  1938.3, 3825.4) |
| GMFR  (95% CI)^c^ | 7.2  (5.7, 9.0) | 7.8  (5.9, 10.4) |
| Seroresponse^e^, n (%)^f^ (95% CI)^g^ | 55 (73.3)  (61.9, 82.9) | 48 (69.6)  (57.3, 80.1) |
| **Day 91** | | |
| n^b^ | 73 | 66 |
| GMT  (95% CI)^c^ | 978.5  (667.7, 1434.0) | 1713.5  (1238.2, 2371.4) |
| GMFR  (95% CI)^c^ | 4.6  (3.3, 6.3) | 5.0  (3.6, 6.8) |
| Seroresponse^e^, n (%)^f^ (95% CI)^g^ | 32 (43.8)  (32.2, 55.9) | 35 (53.0)  (40.3, 65.4) |
| **Day 181** | | |
| n^b^ | 73 | 64 |
| GMT  (95% CI)^c^ | 724.0  (500.9, 1046.5) | 1608.7  (1053.9, 2455.5) |
| GMFR  (95% CI)^c^ | 3.4  (2.3, 5.0) | 4.5  (2.8, 7.3) |
| Seroresponse^e^, n (%)^f^ (95% CI)^g^ | 26 (35.6)  (24.7, 47.7) | 28 (43.8)  (31.4, 56.7) |
| **Day 366** | | |
| n^b^ | 43 | 39 |
| GMT  (95% CI)^c^ | 666.0  (395.5, 1121.8) | 1873.7  (1156.9, 3034.9) |
| GMFR  (95% CI)^c^ | 2.5  (1.5, 4.3) | 6.4  (3.3, 12.6) |
| Seroresponse^e^, n (%)^f^ (95% CI)^g^ | 20 (46.5)  (31.2, 62.3) | 24 (61.5)  (44.6, 76.6) |

The LLOQ was 18.5 for the pseudovirus nAb ID_50_ titers against SARS-CoV-2 D614G, and 19.85 for titers against Omicron BA.1. Numbers below the LLOQ were replaced by 0.5 × LLOQ.

^a^The PPIS-Neg consisted of participants in the PPIS who were pre booster SARS-CoV-2 negative, defined as no virologic or serological evidence of SARS-CoV-2 infection on or before booster, ie, RT-PCR result was not positive if available pre-booster and a negative bAb specific to SARS-CoV-2 nucleocapsid on or before booster.

^b^Number of participants with non-missing nAb data at the timepoint (baseline or post-baseline).

^c^95% CI is calculated based on the t-distribution of the log-transformed values or the difference in the log-transformed values for GMT and GMFR, respectively, then back transformed to the original scale for presentation.

^d^Percentages are based on n.

^e^Pre-booster seroresponse at a participant level is defined as an increase from below the LLOQ to ≥4 × LLOQ if pre-booster baseline nAb titer is <LLOQ, or at least a 4-fold rise if pre-booster baseline nAb titer is ≥LLOQ.

^f^Number of participants meeting the criterion at the time point. Percentage is based on number of participants with non-missing data at baseline and the corresponding post-baseline time point.

^g^95% CI is calculated using the Clopper-Pearson method.

*Abbreviations*: bAb, binding antibody; CI, confidence interval; GM, geometric mean; GMFR, geometric mean fold rise; GMT, geometric mean titer; LLOQ, lower limit of quantification; mRNA, messenger RNA; nAb, neutralizing antibody; PPIS, Per-Protocol Immunogenicity Set; PPIS-Neg, Per-Protocol Immunogenicity Set SARS-CoV-2–negative; RT-PCR, reverse transcription-polymerase chain reaction.

## Table S9. Summary of neutralizing antibody responses at all time points by SARS-CoV-2 variant in Part B (PPIS^a^) – sensitivity analysis

| Time Point | **mRNA-1283.529** | |
| --- | --- | --- |
|  | **5 µg**  **(N = 100)** | **10 µg**  **(N = 94)** |
| **SARS-CoV-2 D614G** | | |
| **Baseline (Day 1)** | | |
| n^b^ | 100 | 94 |
| GMT  (95% CI)^c^ | 1718.0  (1303.0, 2265.3) | 2218.8  (1753.0, 2808.2) |
| Participants ≥LLOQ, n (%)^d^ | 100 (100) | 94 (100) |
| **Day 29** | | |
| n^b^ | 100 | 94 |
| GMT  (95% CI)^c^ | 3878.1  (3093.3, 4862.1) | 5413.7  (4315.8, 6790.9) |
| GMFR  (95% CI)^c^ | 2.3 (1.9, 2.6) | 2.4 (2.1, 2.9) |
| Seroresponse^e^, n (%)^f^ (95% CI)^g^ | 16 (16.0)  (9.4, 24.7) | 17 (18.1)  (10.9, 27.4) |
| **Day 91** | | |
| n^b^ | 97 | 90 |
| GMT  (95% CI)^c^ | 2565.6  (1989.7, 3308.1) | 3571.7  (2847.6, 4479.9) |
| GMFR  (95% CI)^c^ | 1.5 (1.2, 1.8) | 1.6 (1.4, 1.9) |
| Seroresponse^e^, n (%)^f^ (95% CI)^g^ | 11 (11.3)  (5.8, 19.4) | 10 (11.1)  (5.5, 19.5) |
| **Day 181** | | |
| n^b^ | 98 | 89 |
| GMT  (95% CI)^c^ | 1829.5  (1415.0, 2365.3) | 2500.4  (1933.6, 3233.3) |
| GMFR  (95% CI)^c^ | 1.1 (0.8, 1.4) | 1.1 (0.9, 1.4) |
| Seroresponse^e^, n (%)^f^ (95% CI)^g^ | 18 (18.4) (11.3, 27.5) | 14 (15.7)  (8.9, 25.0) |
| **Day 366** | | |
| n^b^ | 60 | 55 |
| GMT  (95% CI)^c^ | 1618.7  (1124.2, 2330.9) | 2179.7  (1539.8, 3085.6) |
| GMFR  (95% CI)^c^ | 0.8 (0.6, 1.2) | 1.0 (0.6, 1.4) |
| Seroresponse^e^, n (%)^f^ (95% CI)^g^ | 10 (16.7)  (8.3, 28.5) | 11 (20.0)  (10.4, 33.0) |
| **Omicron BA.1** | | |
| **Baseline (Day 1)** | | |
| n^b^ | 100 | 94 |
| GMT  (95% CI)^c^ | 344.4  (248.5, 477.3) | 537.0  (377.0, 764.9) |
| Participants ≥LLOQ, n (%)^d^ | 97 (97.0) | 94 (100) |
| **Day 29** | | |
| n^b^ | 100 | 94 |
| GMT  (95% CI)^c^ | 2219.5  (1627.6, 3026.8) | 3520.0  (2606.4, 4753.7) |
| GMFR  (95% CI)^c^ | 6.4  (5.2, 8.0) | 6.6  (5.2, 8.3) |
| Seroresponse^e^, n (%)^f^ (95% CI)^g^ | 64 (64.0)  (53.8, 73.4) | 62 (66.0)  (55.5, 75.4) |
| **Day 91** | | |
| n^b^ | 97 | 90 |
| GMT  (95% CI)^c^ | 1315.3  (944.7, 1831.3) | 2186.9  (1626.4, 2940.6) |
| GMFR  (95% CI)^c^ | 3.9  (2.9, 5.3) | 4.2  (3.3, 5.3) |
| Seroresponse^e^, n (%)^f^ (95% CI)^g^ | 38 (39.2)  (29.4, 49.6) | 41 (45.6)  (35.0, 56.4) |
| **Day 181** | | |
| n^b^ | 98 | 88 |
| GMT  (95% CI)^c^ | 883.8  (653.6, 1195.1) | 1923.2  (1359.2, 2721.3) |
| GMFR  (95% CI)^c^ | 2.6  (1.8, 3.6) | 3.5  (2.4, 5.1) |
| Seroresponse^e^, n (%)^f^ (95% CI)^g^ | 29 (29.6)  (20.8, 39.7) | 31 (35.2)  (25.3, 46.1) |
| **Day 366** | | |
| n^b^ | 60 | 55 |
| GMT  (95% CI)^c^ | 869.0  (579.1, 1304.0) | 1982.0  (1373.8, 2859.4) |
| GMFR  (95% CI)^c^ | 2.2  (1.3, 3.5) | 3.8  (2.2, 6.6) |
| Seroresponse^e^, n (%)^f^ (95% CI)^g^ | 24 (40.0)  (27.6, 53.5) | 26 (47.3)  (33.7, 61.2) |

The LLOQ was 18.5 for the pseudovirus nAb ID_50_ titers against SARS-CoV-2 D614G, and 19.85 for titers against Omicron BA.1. Numbers below the LLOQ were replaced by 0.5 × LLOQ.

^a^The PPIS consisted of all enrolled participants who received the planned study vaccine, had pre-booster and Day 29 nAb data against prototype virus strain, no previous HIV infection, and no major protocol deviations that impacted key or critical data.

^b^Number of participants with non-missing nAb data at the time point (baseline or post-baseline).

^c^95% CI is calculated based on the t-distribution of the log-transformed values or the difference in the log-transformed values for GMT and GMFR, respectively, then back transformed to the original scale for presentation.

^d^Percentages are based on n.

^e^Pre-booster seroresponse at a participant level is defined as an increase from below the LLOQ to ≥4 × LLOQ if pre-booster baseline nAb titer is <LLOQ, or at least a 4-fold rise if pre-booster baseline nAb titer is ≥LLOQ.

^f^Number of participants meeting the criterion at the time point. Percentage is based on number of participants with non-missing data at baseline and the corresponding post-baseline time point.

^g^95% CI is calculated using the Clopper-Pearson method.

*Abbreviations*: CI, confidence interval; GM, geometric mean; GMFR, geometric mean fold rise; GMT, geometric mean titer; LLOQ, lower limit of quantification; mRNA, messenger RNA; nAb, neutralizing antibody; PPIS, Per-Protocol Immunogenicity Set.

## Table S10. Summary of SARS-CoV-2 infections and COVID-19 cases detected starting ≥14 days after vaccination throughout the study in Part A (Full Analysis Set)

| **n, (%)** | **mRNA-1283** | | | **mRNA-1283.211** | | **mRNA-1273** |
| --- | --- | --- | --- | --- | --- | --- |
|  | 2.5 µg  (**N** = 57) | 5 µg  (**N** = 63) | 10 µg  (**N** = 56) | 5 µg  (**N** = 53) | 10 µg  (**N** = 54) | 50 µg  (**N** = 57) |
| SARS-CoV-2 infection,^a,b^ n (%)  [Number of events] | 21 (36.8)  [21] | 26 (41.3)  [26] | 21 (37.5)  [21] | 17 (32.1)  [17] | 14 (25.9)  [14] | 15 (26.3)  [15] |
| Asymptomatic SARS-CoV-2 infection,^c^ n (%)  [Number of events] | 15 (26.3)  [15] | 19 (30.2)  [19] | 12 (21.4)  [12] | 9 (17.0)  [9] | 10 (18.5)  [10] | 7 (12.3)  [7] |
| COVID-19, primary case definition,^d^ n (%)  [Number of events] | 7 (12.3)  [7] | 5 (7.9)  [5] | 14 (25.0)  [15] | 10 (18.9)  [10] | 9 (16.7)  [10] | 10 (17.5)  [11] |
| COVID-19, secondary case definition,^e^ n (%)  [Number of events] | 8 (14.0)  [8] | 7 (11.1)  [7] | 14 (25.0)  [16] | 11 (20.8)  [11] | 9 (16.7)  [10] | 10 (17.5)  [11] |

Percentage is based on the number of participants in Full Analysis Set (all randomized participants who received study vaccine).

^a^SARS-CoV-2 infection: includes both symptomatic and asymptomatic infections; cases are defined in participants with negative SARS-CoV-2 status at pre-booster by either positive post-baseline bAb levels against SARS-CoV-2 nucleocapsid protein or a positive post-baseline RT-PCR test result.

^b^Symptomatic SARS-CoV-2 infection: cases are defined in participants with signs and symptoms meeting the US Centers for Disease Control and Prevention (CDC) case definition and the case definition from the Phase 3 study (mRNA-1273-P301) for COVID-19, as well as the clinical suspicion of the site investigator.

^c^Asymptomatic SARS-CoV-2 infection: cases are defined in participants with negative SARS-CoV-2 status at pre-booster and absence of COVID-19 symptoms by either positive post-baseline bAb levels against SARS-CoV-2 nucleocapsid protein or a positive post-baseline RT-PCR test result.

^d^Primary case definition of COVID-19: cases are defined as fulfilling clinical criteria based on both symptoms of COVID-19 (≥2 systemic or ≥1 respiratory symptom) and a positive RT-PCR test result.

^e^Secondary case definition of COVID-19: cases are defined as fulfilling clinical criteria based on both symptoms of COVID-19 (≥1 CDC-listed systemic or respiratory symptom) and a positive RT-PCR test result.

## Table S11. Summary of SARS-CoV-2 infections and COVID-19 cases detected starting ≥14 days after vaccination throughout the study in Part B (Full Analysis Set)

| **n, (%)** | **mRNA-1283.529** | |
| --- | --- | --- |
|  | 5 µg  (**N** = 103) | 10 µg  (**N** = 97) |
| SARS-CoV-2 infection,^a,b^ n (%)  [Number of events] | 36 (35.0)  [36] | 47 (48.5)  [47] |
| Asymptomatic SARS-CoV-2 infection,^c^ n (%)  [Number of events] | 12 (11.7)  [12] | 11 (11.3)  [11] |
| COVID-19, primary case definition,^d^ n (%)  [Number of events] | 28 (27.2)  [29] | 36 (37.1)  [38] |
| COVID-19, secondary case definition,^e^ n (%)  [Number of events] | 28 (27.2)  [29] | 42 (43.3)  [44] |

Percentage is based on the number of participants in Full Analysis Set (all enrolled participants who received study vaccine).

^a^SARS-CoV-2 infection: includes both symptomatic and asymptomatic infections; cases are defined in participants with negative SARS-CoV-2 status at pre-booster by either positive post-baseline bAb levels against SARS-CoV-2 nucleocapsid protein or a positive post-baseline RT-PCR test result.

^b^Symptomatic SARS-CoV-2 infection: cases are defined in participants with signs and symptoms meeting the US Centers for Disease Control and Prevention (CDC) case definition and the case definition from the Phase 3 study (mRNA-1273-P301) for COVID-19, as well as the clinical suspicion of the site investigator.

^c^Asymptomatic SARS-CoV-2 infection: cases are defined in participants with negative SARS-CoV-2 status at pre-booster and absence of COVID-19 symptoms by either positive post-baseline bAb levels against SARS-CoV-2 nucleocapsid protein or a positive post-baseline RT-PCR test result.

^d^Primary case definition of COVID-19: cases are defined as fulfilling clinical criteria based on both symptoms of COVID-19 (≥2 systemic or ≥1 respiratory symptom) and a positive RT-PCR test result.

^e^Secondary case definition of COVID-19: cases are defined as fulfilling clinical criteria based on both symptoms of COVID-19 (≥1 CDC-listed systemic or respiratory symptom) and a positive RT-PCR test result.
